# FLAME: Federated Learning and Aggregated Multi-Model Ensemble for Multi-Class Alzheimer’s Disease Stage Classification from Structured Clinical Data

**DOI:** 10.3390/diagnostics16132029

**Published:** 2026-06-29

**Authors:** Karim Gasmi, Lassaad Ben Ammar, Moez Krichen, Ahod Alghuried

**Affiliations:** 1 Department of Computer Science, College of Computer and Information Sciences, Jouf University, Sakaka 72388, Saudi Arabia; kgasmi@ju.edu.sa; 2 Department of Computer Sciences, College of Computer Engineering and Sciences, Prince Sattam bin Abdulaziz University, Al-Kharj 11942, Saudi Arabia; a.alghareed@psau.edu.sa; 3 Faculty of Computing and Information, Al-Baha University, Al-Baha 65528, Saudi Arabia; pr.moez.k@redcad.org; 4 ReDCAD Laboratory, University of Sfax, Sfax 3038, Tunisia

**Keywords:** SDG 3, Alzheimer’s disease classification, hybrid ensemble learning, deep neural network, weight optimisation, clinical tabular data, privacy-preserving machine learning, multi-class classification

## Abstract

**Background/Objectives**: The precise identification of Alzheimer’s disease (AD) stages through clinical data is crucial for early diagnosis and suitable therapy. This classification remains troublesome due to overlap in cognitive profiles across different phases of illness progression. This study presents a comprehensive and advanced diagnostic system, termed FLAME, featuring an enhanced federated learning architecture for privacy-preserving multi-institutional implementation. It provides a systematic review of machine learning (ML) and deep learning (DL) models for the classification of five stages of Alzheimer’s disease (AD). The models include cognitively normal (CN), subjective memory complaints (SMC), early mild cognitive impairment (EMCI), late mild cognitive impairment (LMCI), and Alzheimer’s disease (AD). **Methods**: Sixteen traditional machine learning models and eleven deep learning architectures—including FT-Transformer and NODE—were evaluated using a structured clinical dataset comprising 362 features. A hybrid ensemble was created at the probability level by combining the two top-performing models, LightGBM and a five-layer DNN. The weights of this ensemble were automatically optimised using a Genetic Algorithm (GA) with Macro-F1 as the fitness criterion, confirmed stable across 30 independent runs ($w^{★}=0.5024\pm 0.0001$). A federated learning architecture was then established, deploying the DNN across non-IID clients while keeping LightGBM centralised. We examine four distinct aggregation algorithms: FedAvg, FedProx, FedNova, and SCAFFOLD. **Results**: Among all deep learning architectures, FT-Transformer achieved the highest standalone performance (accuracy = 0.7810, κ = 0.7081). The five-layer deep neural network (DNN) was selected as the DL representative for the hybrid ensemble. LightGBM attained superior machine learning performance (accuracy = 0.8156, κ = 0.7537), confirmed deterministic across 10 seeds. The LightGBM vs. XGBoost difference is not statistically significant (McNemar p=0.4227). The GA-optimised hybrid ensemble (w = 0.685) surpassed both individual baselines across all evaluation metrics. The FedNova hybrid design achieved superior overall performance in federated configurations, surpassing all centralised arrangements in accuracy (accuracy = 0.8213, κ 0.7614). **Conclusions**: Evolutionary ensemble optimisation combined with federated learning provides a robust, scalable, and privacy-preserving solution for AD stage classification, offering a clinically viable framework for real-world multi-institutional decision-support systems. However, the AD class remains severely under-recalled across all configurations (F1 ≤ 0.21), identifying this as the primary open challenge for clinical translation.

## 1. Introduction

Alzheimer’s disease (AD) is the predominant aetiology of dementia globally. Alzheimer’s disease is a gradual, irreversible neurological condition. It is the predominant cause of dementia overall. A characteristic of this disease is the gradual deterioration of cognitive functioning, including memory, thinking, and executive skills. It ultimately results in a loss of autonomy and constitutes a significant financial burden for patients, carers, and healthcare systems. Consequently, timely and accurate diagnosis of the stage of Alzheimer’s disease is crucial for early intervention, enhanced disease management, and effective clinical decision-support systems.

The clinical progression of Alzheimer’s disease is sometimes characterised as a continuum that includes cognitively normal individuals, those exhibiting intermediate cognitive impairment, and individuals with fully manifested Alzheimer’s disease. Mild cognitive impairment (MCI) is a significant element in this process. MCI is typically categorised into early MCI (EMCI) and late MCI (LMCI), indicating the progressive severity of cognitive deterioration. Certain clinical datasets include a category termed Subjective Memory Complaints (SMC). This group comprises individuals who report memory issues yet have no measurable cognitive deficits. This detailed categorisation facilitates precise clinical labelling but also leads to significant diagnostic ambiguity, especially within the CN and SMC groups, whose cognitive and clinical traits frequently intersect. This is particularly applicable to the two groups.

In recent years, machine learning (ML) techniques have shown considerable potential for classifying Alzheimer’s disease using structured clinical and biomarker data. Conventional machine learning techniques are widely used for tabular data due to their reliability, interpretability, and effectiveness. Models of this nature include logistic regression, support vector machines, random forests, gradient boosting, XGBoost, and LightGBM. Ensemble-based methodologies have demonstrated superior performance by effectively capturing non-linear correlations and extensive feature interactions. Nonetheless, conventional machine learning techniques may remain constrained in simulating higher-level associations when the diagnostic categories exhibit substantial overlap.

Deep learning methodologies have considerably broadened the scope of Alzheimer’s disease modelling by enabling adaptable, non-linear representations that can discern intricate data patterns. In this area, numerous neural designs have been examined. These designs encompass fully connected networks, convolutional architectures, recurrent architectures, and residual tabular networks. Nonetheless, implementing deep learning models on tabular clinical data presents numerous challenges, including limited sample sizes, overfitting, and lengthy hyperparameter optimisation. This is despite the success of these models in imaging applications. Consequently, their performance does not consistently exceed that of well-structured ensembles of machine learning algorithms.

Significant advancements have occurred; nonetheless, gaps remain in the existing literature. Initially, some studies concentrate on the classification of Alzheimer’s disease utilising binary or fixed multi-class methodologies. Nonetheless, these experiments do not assess the impact of diagnostic ambiguity on the model’s performance. Secondly, machine learning and deep learning methodologies are typically examined separately rather than inside a cohesive comparative framework. Third, ensemble learning is widely utilised; however, most studies employ fixed or heuristic weighting procedures, thereby hindering the effective exploitation of model complementarity. In conclusion, although incorporating privacy-preserving federated learning is crucial for practical clinical implementation across numerous institutions, it remains largely unexamined in frameworks for Alzheimer’s disease classification.

This study presents a systematic and progressive approach to classifying the stages of Alzheimer’s disease using clinical tabular data. The primary objective of this study is to rectify the identified deficiencies. We create a classification challenge comprising five classes to evaluate the model’s performance throughout the entire cognitive spectrum. This issue encompasses CN, SMC, EMCI, LMCI, and AD. A comprehensive comparison is conducted among sixteen classical machine learning models and eleven deep learning architectures, including NODE and FT-Transformer. The most effective machine learning model is LightGBM, although the five-layer deep neural network (DNN) performs best among deep learning models. A hybrid ensemble model is proposed based on these observations. This model integrates the two most effective paradigms: LightGBM and a five-layer DNN. The optimal ensemble weights are automatically ascertained by a Genetic Algorithm, utilising Macro-F1 as the fitness metric. This facilitates the implementation of an adaptive and data-driven fusion methodology. To enhance the deployment framework while safeguarding user privacy, we introduce a federated learning architecture. The DNN component of this design is disseminated across non-IID clients, whereas LightGBM is centralised. The weight optimised by GA establishes the composition of these components for four distinct aggregation algorithms: FedAvg, FedProx, FedNova, and SCAFFOLD.

The main contributions of this study can be summarised as follows:

- A systematic and unified comparison of sixteen classical machine learning models and eleven deep learning architectures (including NODE and FT-Transformer) on a five-class Alzheimerś disease classification task, using a leakage-free predefined split and a comprehensive set of evaluation metrics.
- Empirical identification of LightGBM as the best classical ML model and FT-Transformer as the best standalone deep learning architecture (Acc = 0.7810). The DNN (5-layer) is retained as the DL representative in the hybrid ensemble due to its superior federated stability, validated by an ablation study.
- A GA-optimised hybrid ensemble framework that adaptively combines the best ML and DL representatives through evolutionary weight search, consistently outperforming individual models across all metrics.
- A federated learning architecture that distributes the DNN component across non-IID clients while maintaining LightGBM centrally, integrated via the GA-derived optimal weight, achieving the best overall result with FedNova and surpassing all centralised configurations.

The remainder of this document is structured as follows. Section 2 presents a comprehensive literature review on the stages of Alzheimer’s disease, traditional machine learning and deep learning classification techniques, ensemble methodologies, and federated learning approaches for clinical data. Section 3 delineates the dataset, the preprocessing pipeline, the comprehensive range of classical machine learning and deep learning architectures evaluated, the development of the GA-optimised hybrid ensemble, and the federated learning framework. Section 4 delineates and analyses the experimental findings. This section comprises four subsections: a comparison of classical machine learning models (Section 4.1), a comparison of deep learning architectures (Section 4.2), an evaluation of GA-optimised hybrid ensembles (Section 4.3), and an evaluation of federated learning with hybrid ensembles (Section 4.4). Section 4.6 delineates the existing constraints of the proposed framework and offers a synopsis of prospective research avenues. The final section of the study, designated as Section 5, provides a comprehensive summary of the principal findings and their significance in the medical field.

## 2. Related Work

This section reviews the literature underpinning the three methodological pillars of the proposed framework: clinical staging of Alzheimer’s disease, classical and deep learning approaches to AD classification, and federated learning for privacy-preserving medical data analysis. For each pillar, we identify the key contributions, highlight persistent limitations, and position the present work within the broader research landscape.

### 2.1. Alzheimer’s Disease: Clinical Background and Staging Frameworks

The WHO study on dementia in the 21st century [1,2] indicates that Alzheimer’s disease (AD) is the predominant form of dementia globally and poses a significant public health challenge in this century. The disease is marked by the progressive accumulation of amyloid-beta plaques and neurofibrillary tau tangles, leading to synaptic dysfunction, neuronal death, and ultimately permanent cognitive decline [3,4,5]. The progression of Alzheimer’s disease (AD) ranges from cognitively normal (CN) to mild cognitive impairment (MCI), ultimately culminating in advanced dementia [6,7].

The concept of moderate cognitive impairment (MCI) was initially formalised by Petersen et al. It has now become a cornerstone of pre-dementia research. This concept denotes a phase between typical ageing and dementia, characterised by noticeable cognitive impairments that do not yet hinder daily functioning. The classification of MCI into early MCI (EMCI) and late MCI (LMCI) reflects the differences in the severity of episodic memory deficits and the rate of progression to Alzheimer’s disease (AD) [8,9]. Subsequently, the endeavour culminated in the definitive division of MCI into these two classifications. Longitudinal studies indicate that persons with LMCI transition to Alzheimer’s disease at a significantly higher incidence than those with EMCI, leading to initiatives aimed at developing a more nuanced classification.

The notion of Subjective Memory Complaints (SMC), referred to as Subjective Cognitive Decline (SCD) in different contexts, has garnered interest as a potential precursor to mild cognitive impairment (MCI) [10,11]. Longitudinal studies indicate a heightened amyloid burden and an elevated long-term risk of advancing to moderate cognitive impairment or Alzheimer’s disease in individuals with subjective memory complaints [12,13]. Individuals with SMC report experiencing memory issues without any discernible outward deficits. Nonetheless, a significant overlap exists between the cognitive and biomarker profiles of individuals with Subjective Memory Complaints (SMC) and those classified as Cognitively Normal (CN), resulting in persistent hurdles for automated classification systems [10]. Nevertheless, most previous studies did not systematically examine the effect of including or excluding SMC categories on classification performance. This is a significant deficiency in the current body of research.

The Alzheimer’s Disease Neuroimaging Initiative (ADNI), launched in 2004, has been pivotal in facilitating extensive multi-site research. This has been accomplished by supplying standardised longitudinal data comprising neuroimaging, cognitive assessments, cerebrospinal fluid (CSF) biomarkers, and genetic information [14,15]. The majority of the machine learning studies examined in this study utilised ADNI data. Nonetheless, inconsistencies in cohort selection, preprocessing methodologies, and class definitions render direct comparisons among the numerous studies challenging.

### 2.2. Classical Machine Learning for Alzheimer’s Disease Classification

A substantial body of research has been conducted with conventional supervised machine learning techniques to address the diagnosis and staging of Alzheimer’s disease (AD), utilising structured clinical and biomarker data. Support vector machines (SVMs) and morphological data from magnetic resonance imaging (MRI) have shown high accuracy in differentiating between Alzheimer’s disease (AD) and congenital neural networks (CNs) [16,17]. Support vector machines (SVMs) are among the earliest and most widely used machine learning methodologies. Logistic regression has been employed as an interpretable baseline in numerous investigations. Refs. [18,19] demonstrate that it attains competitive accuracy on well-organised datasets and offers clear feature weighting.

In recent years, random forests have garnered attention in machine learning for their ability to handle diverse data sources, their resilience to noisy features, and their inherent feature importance estimation [20]. Numerous studies [21,22] demonstrated that random forests outperform support vector machines (SVMs) in multi-class Alzheimer’s disease staging tasks when utilised on heterogeneous tabular data, including neuropsychological scores, demographic characteristics, and biomarkers. Gradient boosting machines (GBMs) [23] and their successors, such as XGBoost [24,25] and LightGBM [26,27], have advanced ensemble-based classification by pioneering efficient gradient-based tree induction with regularisation techniques to mitigate overfitting. XGBoost is the established standard for tabular classification benchmarks, owing to its computational efficiency, capacity to handle missing data, and strong out-of-the-box performance [24,28].

The prediction of MCI conversion [29], multi-class staging [30], and discrimination tasks based on biomarkers [31] have utilised XGBoost and its gradient-boosting variants, using datasets from ADNI. These applications have been implemented for Alzheimer’s disease (AD) classification. Raghavendra et al. [32] and Tong et al. [33] demonstrated that ensemble tree methodologies effectively capture non-linear interactions among clinical factors that simpler models fail to address. It is important to acknowledge that these investigations predominantly employ predetermined hyperparameter configurations or grid search methodologies. Moreover, they neglect to investigate adaptive ensemble weighting or enhance model combination methodologies.

Furthermore, lightweight alternatives such as K-nearest neighbours (KNN) and naïve Bayes classifiers are studied, often serving as comparative baselines, as detailed in the studies by [33,34]. These methods are straightforward and facilitate rapid inference; nevertheless, they typically produce inferior results compared to more expressive models when dealing with the overlapping class distributions commonly found in multi-stage AD datasets. A notable deficiency of this work is that classical models are assessed in isolation rather than within a cohesive framework that systematically evaluates multiple paradigms on the same dataset and task design.

### 2.3. Deep Learning Approaches for Alzheimer’s Disease Diagnosis

The way Alzheimer’s disease (AD) is detected has been dramatically changed by deep learning, particularly by analysing neuroimaging data. Convolutional neural networks (CNNs) [35] have demonstrated human-competitive performance in the classification of Alzheimer’s disease (AD) versus congenital neurone (CN) and in the prediction of MCI conversion when applied on structural magnetic resonance imaging (MRI) [36,37,38]. Three-dimensional convolutional neural network (CNN) architectures that can process whole-brain volumetric images have enabled the extraction of spatially localised atrophy patterns without manual feature engineering [39,40]. As previously mentioned in the works of Hon (2017) and Ebrahimighahnavieh (2020) in [41,42], transfer learning procedures (i.e., the adaptation of models pre-trained on large natural image datasets) have been shown to be particularly effective in alleviating the restricted sample-size problem that is inherent to medical imaging.

Longitudinal clinical trajectories have been modelled with recurrent neural networks (RNNs) and long short-term memory (LSTM) networks. Such networks have been used to model temporal dependencies in repeated cognitive tests and biomarker measures [43,44]. The approaches are particularly suited to ADNI’s longitudinal study design, allowing for the prediction of future cognitive states from historical sequences. Graph neural networks (GNNs) have broadened the representation reach by representing structural and functional brain connectivity as graphs. This has enabled information propagation across anatomically connected regions [45,46].

More recently, transformer-based architectures [47], originally introduced for natural language processing, have been adapted for medical imaging and multimodal clinical data. Vision transformers (ViTs) [48] and their variants have achieved competitive performance in brain MRI classification by leveraging self-attention approaches to capture global spatial dependencies beyond the receptive-field limits of CNNs [49]. This was achieved by exploiting the powers of vision transformers. Furthermore, multimodal fusion frameworks incorporating imaging, genetic, and clinical data have been proposed, which leverage complementary information across modalities using cross-attention or late-fusion transformer architectures [50].

Despite these advances, deep learning models applied to structured tabular clinical data (as opposed to pictures) suffer from well-documented issues. Deep learning on low-dimensional clinical tables is less robust compared to other approaches because of the small size of the datasets, a high risk of overfitting, and a large sensitivity to hyperparameters [51,52]. It is interesting to note that systematic benchmarking studies by Grinsztajn et al. [52] and Shwartz-Ziv and Armon [51] demonstrated that gradient boosting ensembles consistently deliver performance equal to or better than deep learning architectures on tabular benchmarks. These results challenge the premise that deep learning supremacy is consistent across data modalities.

### 2.4. Deep Learning Architectures Tailored for Tabular Data

A substantial body of research has shown that systems specifically engineered to handle structured data perform better. This study is motivated by the inadequate performance of conventional deep neural networks on tabular data. TabNet, introduced by Arik and Pfister [53], employs sequential attention mechanisms to select sparse subsets of features at each decision point. This enables the simultaneous generation of interpretable feature significance maps and competitive classification accuracy. NODE (Neural Oblivious Decision Ensembles) is a framework that generalises gradient-boosted trees within a differentiable neural architecture [54]. It achieves this by layering oblivious decision trees, facilitating end-to-end optimisation by backpropagation.

The FT-Transformer, or Feature Tokeniser Transformer [55], demonstrated that transformer designs can be effective for tabular data by tokenising individual features into embeddings and applying multi-head self-attention to the feature tokens. Gorishniy et al. [55] demonstrated that the FT-Transformer algorithm outperformed prior tabular deep learning techniques across multiple benchmarks and was competitive with gradient boosting. The SAINT (Self-Attention and Intersample Attention Transformer) [56] further advanced this concept by integrating both within-sample and between-sample attention mechanisms. It utilised contrastive self-supervised pre-training to enhance generalisation with minimal monitoring.

The Tabular Prior-Data Fitted Network (TabPFN), first introduced by Hollmann and colleagues [57], is one of the most remarkable innovations of recent years. TabPFN is a transformer trained offline on millions of synthetic microtabular classification tasks derived from a probabilistic prior over datasets. This contrasts with typical models that require fitting to the target data. For inference, TabPFN carries out a singular forward pass to estimate the Bayesian posterior. The estimation relies on the provided contextual training data, with no gradient modifications applied. Due to its in-context learning paradigm [58], TabPFN demonstrates effective generalisation on datasets including about one thousand samples, a scenario frequently encountered in clinical research. Hollmann et al. [57] indicated that TabPFN surpasses numerous conventional and deep learning baselines on small tabular benchmarks while significantly decreasing inference time. TabPFN v2 [59] is an enhanced version of TabPFN that broadens its applicability to larger datasets and regression tasks, thereby improving its utility in the medical field. However, TabPFN has been minimally evaluated for neurodegenerative disease categorisation, and its synergy with boosting-based techniques in hybrid ensembles remains unexamined.

### 2.5. Multimodal and Feature Engineering Approaches

A substantial body of research has focused on feature engineering and multimodal data integration for the categorisation of Alzheimer’s disease, including the selection of model architecture. The Mini-Mental State Examination (MMSE), the Alzheimer’s Disease Assessment Scale-Cognitive Subscale (ADAS-Cog), the Clinical Dementia Rating Sum of Boxes (CDR-SB), and the Rey Auditory Verbal Learning Test (RAVLT) are neuropsychological assessments that have consistently been recognised as highly discriminative indicators for staging tasks [60]. MRI measurements, including hippocampus volume and entorhinal cortex thickness, provide complementary structural indicators of neurodegeneration [61,62]. Furthermore, these values are derived via magnetic resonance imaging (MRI) technology.

The apolipoprotein E epsilon-4 allele (APOE-ε4) has been incorporated as a binary or dosage-coded covariate in classification models, owing to its known association with the risk of Alzheimer’s disease [63,64]. Amyloid-beta 1-42 (A$\beta_{42}$), total tau, and phosphorylated tau are cerebrospinal fluid (CSF) biomarkers indicative of fundamental pathological processes. These biomarkers have demonstrated significant predictive efficacy in supervised classification contexts, as evidenced by the research of Hansson (2018) and Blennow (2010) in [65,66]. Recursive feature elimination, LASSO regularisation, and mutual information filtering [67,68] are prominent feature selection techniques used to reduce dimensionality and improve generalisation when the number of available features exceeds the number of samples.

A number of efforts have been undertaken to develop multimodal fusion frameworks. These frameworks amalgamate imaging, genetic, cognitive, and cerebrospinal fluid (CSF) data to leverage the complementary information provided by each modality [18,21,69]. Multimodal approaches sometimes surpass unimodal methods; however, they often encounter challenges such as data incompleteness, modality alignment issues, and increased model complexity. This study addresses structured clinical tabular data devoid of specific imaging elements. This aims to replicate authentic deployment settings in which neuroimagining may not be readily available.

### 2.6. Ensemble Learning and Model Combination Strategies

Ensemble approaches amalgamate the predictions of numerous base learners [70,71,72] to achieve enhanced accuracy, robustness, and diversity. Ensembles are recognised for reducing variance while maintaining a low bias by averaging numerous models that are only marginally accurate [73]. This is the theoretical foundation of ensemble learning. Each of the three paradigms possesses distinct mechanisms for producing and amalgamating variety. The three primary principles are bagging [73], boosting [23,74], and stacking [75].

In research on Alzheimer’s disease (AD) classification, ensemble techniques have been employed across several modalities and task formulations [76]. Integrating predictions from models trained on diverse feature subsets or data partitions has been accomplished by majority voting and basic averaging methods [21,77]. Weighted averaging methods have demonstrated superior performance compared to uniform weighting [78]. These systems assess each model’s contribution based on its anticipated performance. Stacking techniques are utilised to develop a meta-learner that correlates base model outputs with final predictions. This facilitates adaptive combination, though at the cost of increased training complexity [75,79].

Despite advancements, the prevailing methods for constructing ensembles in the AD literature have relied on heuristic or manually calibrated weighting systems. Although evolutionary algorithms have proven effective in various biomedical classification challenges, focus on their application to ensemble optimisation in this domain has been limited. Genetic Algorithms (GAs) [80,81] provide a population-based search methodology that investigates the ensemble weight space through selection, crossover, and mutation operators. The strategy facilitates convergence to globally optimal combinations without requiring gradient information during convergence. Genetic Algorithms (GAs) are extensively employed in the medical domain for feature selection [82], neural architecture search [83], and model hyperparameter optimisation [84]. Nonetheless, the application of Genetic Algorithms to improve the ensemble weights of hybrid classical-deep learning frameworks for Alzheimer’s disease categorisation remains an uncharted territory.

Recent work on AutoML and neural architecture search (NAS) has introduced more systematic approaches to model selection and combination [85,86]; however, these methods typically target individual model optimisation rather than principled cross-paradigm ensemble construction. The combination of XGBoost and TabPFN—representing the best-performing classical and in-context learning paradigms, respectively, within a GA-optimised ensemble—constitutes a novel contribution that addresses the identified gap.

### 2.7. Summary and Research Gaps

The reviewed literature indicates several significant deficiencies that constitute the primary impetus for the current investigation. Despite the clinical importance of fine-grained Alzheimer’s disease staging, the majority of studies employ binary classification methods rather than multi-class classification approaches. Moreover, several studies fail to evaluate the influence of diagnostic ambiguity on model performance. The assessment of traditional machine learning and deep learning methodologies is often conducted in distinct research streams rather than within integrated comparison frameworks. Consequently, the conclusions on the comparative strengths and limitations of the various paradigms are constrained. It is noteworthy that, although ensemble techniques are prevalent, ensembles in the AD classification literature have primarily relied on predetermined or heuristic weighting methods rather than systematic optimisation. The fourth pertains to privacy data acquired using federated learning for Alzheimer’s disease.

This study directly tackles all four deficiencies by (1) systematically analysing the impact of multi-class formulation; (2) offering a thorough cross-paradigm comparison of classical and deep learning models utilising the same clinical dataset; (3) proposing an ensemble weighting strategy based on Genetic Algorithms that adaptively integrates machine learning and deep learning models; (4) empirically validating the efficacy of this hybrid framework for classifying stages of Alzheimer’s disease; and (5) conducting a validation analysis for privacy data based on four federated learning models.

## 3. Materials and Methods

**Figure 1:**
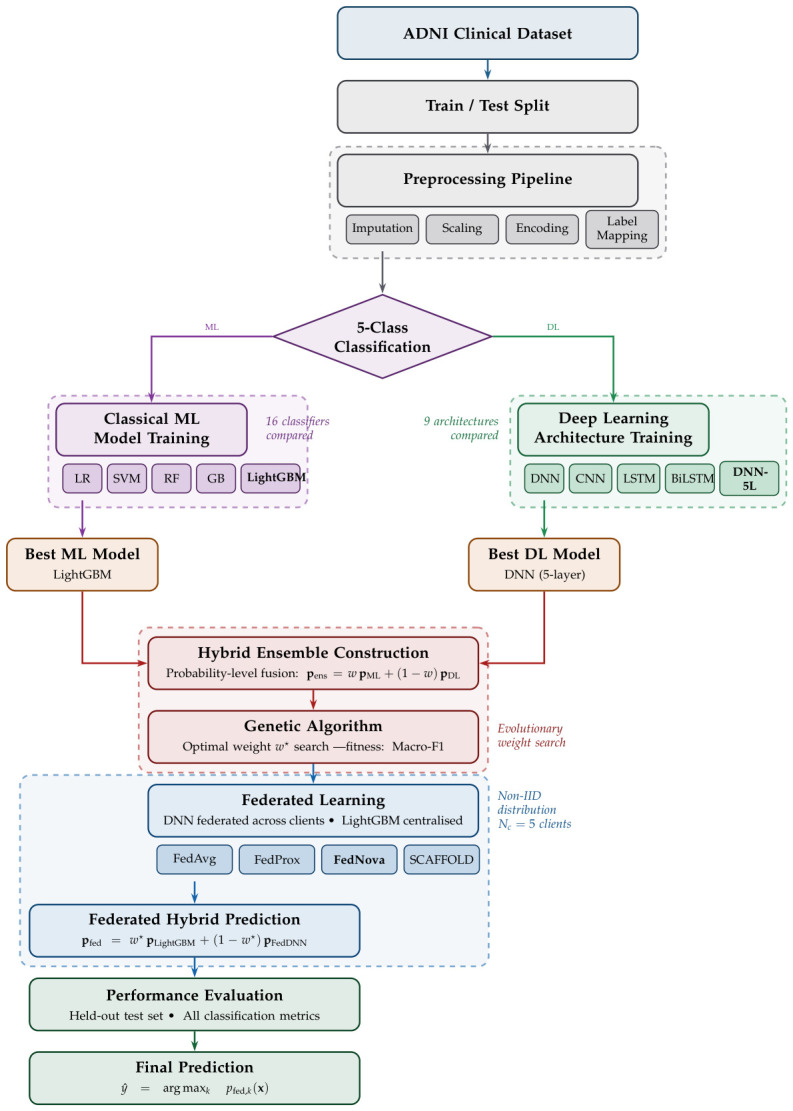
Proposed framework for Alzheimer’s disease stage classification. Starting from the ADNI clinical dataset, a unified preprocessing pipeline is applied before training. Classical ML models and deep learning architectures are compared in parallel under a five-class formulation. The best representatives—LightGBM (ML) and DNN (5-layer) (DL)—are fused via a GA-optimised hybrid ensemble. The DNN component is then distributed across non-IID clients using federated learning (FedAvg, FedProx, FedNova, SCAFFOLD), while LightGBM remains centralised. The GA-derived weight combines both components into the final federated hybrid prediction.

This section outlines the experimental methodology proposed for classifying Alzheimer’s disease stages using structured clinical data. The process commences with a preliminary classification into five categories based on a clinically established formulation. This is followed by an extensive comparison of traditional machine learning and deep learning methodologies to determine the most effective model in each paradigm. The methodology relies on a progressive and modular framework. A hybrid ensemble is formed by amalgamating the top-performing members from each paradigm, with the ensemble weights being automatically optimised by a Genetic Algorithm. Finally, the hybrid ensemble is expanded to a multi-institutional setting via a federated learning framework that safeguards individual privacy. This approach facilitates an open and transparent examination of the model’s activity and enables a reliable assessment of its performance. It also facilitates the logical integration of complementary learning processes. The overall pipeline is illustrated in Figure 1.

### 3.1. Dataset Description and Clinical Variables

This study uses a publicly available structured clinical tabular dataset obtained from the Kaggle platform (https://www.kaggle.com/datasets/sarthakkanjariya/alzheimer-dataset?resource=download (accessed on 12 January 2026)). This dataset is a preprocessed tabular CSV file derived from the Alzheimerś Disease Neuroimaging Initiative (ADNI) cohort and made openly accessible by a third-party contributor on Kaggle. The file contains no individually identifiable information, no raw neuroimaging data, and no biospecimen data. No direct data access agreement with ADNI was required for this study, as the dataset was accessed exclusively through the Kaggle platform in its publicly available form. ADNI is a large-scale, multi-site longitudinal study launched in 2004 that collects standardised neuroimaging, cognitive, biomarker, and genetic data from participants spanning the full spectrum of Alzheimer’s disease progression [14,15]. The dataset contains N=1737 subject-level observations characterised by a heterogeneous collection of clinical, neuropsychological, morphometric, and genetic variables. Each row corresponds to a unique subject and includes a subject identifier, a predefined train/test split indicator (Test_data), a diagnostic label (Diagnosis), and d=362 clinical features.

#### 3.1.1. Feature Groups

The feature set spans five clinically meaningful categories:

##### Cognitive and Neuropsychological Assessments

These include the most discriminative markers of cognitive function routinely collected in ADNI visits:

- **MMSE** (Mini-Mental State Examination): A 30-point scale measuring global cognitive status; lower scores indicate greater impairment.
- **CDRSB** (Clinical Dementia Rating Sum of Boxes): A composite measure of cognitive and functional performance across six domains.
- **ADAS11** and **ADAS13** (Alzheimer’s Disease Assessment Scale–Cognitive Subscale, 11- and 13-item versions): Standardised assessments of memory, language, and praxis; higher scores indicate greater impairment.
- **ADASQ4**: The fourth question of the ADAS addressing delayed word recall.
- **RAVLT** variants (RAVLT_immediate. RAVLT_learning, RAVLT_forgetting. RAVLT_ perc_forgetting): Components of the Rey Auditory Verbal Learning Test quantifying verbal memory encoding, retention, and forgetting.
- **EcogSP** and **EcogPt** subscales: Everyday cognition questionnaire scores from both the study partner and patient perspectives, covering memory, language, visuospatial ability, planning, and organisation.

##### Neuroimaging-Derived Morphometric Measures

Volumetric and thickness measures were derived from T1-weighted structural MRI using automated segmentation:

- **Hippocampus**: Bilateral hippocampal volume (mm^3^), The most established imaging biomarker of AD-related neurodegeneration.
- **WholeBrain**: Total brain parenchymal volume.
- **Entorhinal**: Entorhinal cortex volume, which shows early atrophy in Alzheimer’s disease.
- **Fusiform**: Fusiform gyrus volume, associated with face recognition and semantic memory.
- **MidTemp**: Middle temporal gyrus volume.
- **Ventricles**: Total ventricular volume, which expands as a consequence of brain atrophy.

##### Cerebrospinal Fluid (CSF) Biomarkers

CSF measures reflect core AD pathophysiological processes:

- **ABETA** (Amyloid-$\beta_{1–42}$): Reduced levels indicate amyloid plaque accumulation.
- **TAU**: Total tau protein, elevated in the presence of neurofibrillary tangles and neurodegeneration.
- **PTAU** (phosphorylated tau): A more specific marker of tau pathology.

##### Demographic and Genetic Variables

- **AGE**: Subject age at the time of assessment (years).
- **Year_education**: Years of formal education, used as a proxy for cognitive reserve.
- **Gender**: Biological sex (binary-encoded).
- **APOE4**: Apolipoprotein E ε4 allele dosage (0, 1, or 2 copies), the strongest known genetic risk factor for sporadic AD [63].
- **Ethnicity**, **Race**: Ethnicity and race categories.
- **Marital_status**: Marital status.

##### Derived and Summary Indices

- **FDG**: Mean fluorodeoxyglucose PET uptake, reflecting cerebral metabolic activity.

Table 1 provides a summary of the dataset composition.

#### 3.1.2. Diagnostic Categories and Class Distribution

The original diagnostic labels follow five clinically meaningful categories spanning the Alzheimer’s disease spectrum:

- **CN** (Cognitively Normal): Participants with no subjective or objective cognitive complaints and normal assessment scores.
- **SMC** (Subjective Memory Complaints): Cognitively unimpaired individuals who report perceived memory difficulties without meeting criteria for MCI [10].
- **EMCI** (Early Mild Cognitive Impairment): Individuals with measurable but subtle cognitive deficits that do not significantly impair daily function [8].
- **LMCI** (Late Mild Cognitive Impairment): A more advanced MCI stage with a substantially elevated rate of conversion to AD [87].
- **AD** (Alzheimer’s Disease): Participants meeting diagnostic criteria for probable AD dementia.

This taxonomy aligns with ADNI cohort definitions [14]. SMC participants are recruited from cognitively unimpaired individuals who voluntarily report memory concerns, making them phenotypically proximal to CN subjects and posing a persistent classification challenge for automated systems.

### 3.2. Train–Test Split and Leakage Control

The dataset contains a clear split indicator labelled test data. The training set comprises participants with a test data value of 0 (n = 1390), whereas the held-out test set comprises patients with a test data value of 1 (n = 347). This established subject-level divide guarantees reproducibility and inherently safeguards against data leakage.

All preprocessing transformations (imputation statistics, scaling parameters, categorical encoders) were derived only from the training subset. They were subsequently applied to the test set without any re-evaluation. In classical machine learning models, just the training set was utilised for hyperparameter tuning. This was executed utilising stratified *k*-fold cross-validation (k=5). The reserved test set was used exclusively for reporting the final performance. The fitness criterion was determined using a stratified internal validation split of 80% for training and 20% for validation, with the aim of optimising the ensemble weight via the Genetic Algorithm. This was implemented to ensure the model remained unexposed to the test set during development.

### 3.3. Preprocessing Pipeline

Let $X\in R^{N\times d}$ denote the raw feature matrix and y the corresponding diagnostic labels. The following preprocessing steps were applied sequentially.

#### 3.3.1. Feature Identification and Type Annotation

Features were categorised into continuous (numeric) and categorical types based on their domain and value range. Continuous variables include all cognitive test scores, volumetric measures, CSF biomarkers, age, and education. Categorical variables include sex, ethnicity, race, marital status, and APOE4 allele dosage (treated as ordinal, with 3 discrete levels: 0, 1, 2).

#### 3.3.2. Missing Value Imputation

Missing values were handled separately by feature type:

(1) $$x_{ij}\leftarrow \left\{\begin{array}{cc} x_{ij}, & \mathrm{if}\;\mathrm{observed},\\ \mathrm{median}\left(\{x_{kj}:k\in \mathrm{training}\;\mathrm{set}\}\right), & \mathrm{if}\;\mathrm{missing}\;\mathrm{and}\;\mathrm{continuous},\\ \mathrm{mode}\left(\{x_{kj}:k\in \mathrm{training}\;\mathrm{set}\}\right), & \mathrm{if}\;\mathrm{missing}\;\mathrm{and}\;\mathrm{categorical}. \end{array}\right.$$

Median imputation was preferred over mean imputation for continuous variables because several clinical biomarkers exhibit right-skewed distributions (e.g., CSF amyloid, ventricle volumes). All imputation parameters were derived from the training set and applied identically to the test set.

#### 3.3.3. Standardisation of Continuous Features

Continuous features were standardised using training-set statistics:

(2) $${\tilde{x}}_{ij}=\frac{x_{ij}-\mu_{j}}{\sigma_{j}+\varepsilon },$$

where $\mu_{j}$ and $\sigma_{j}$ are the mean and standard deviation of feature *j* over the training set and $\varepsilon =10^{-8}$ prevents division by zero.

#### 3.3.4. Categorical Encoding

Nominal categorical features were encoded using one-hot encoding. For a feature with *m* distinct categories $\left\{v_{1},\ldots ,v_{m}\right\}$,

$$\mathrm{onehot}\left(v_{k}\right)=e_{k}\in {\left\{0,1\right\}}^{m}.$$

One category per feature was dropped (dummy encoding) to avoid multicollinearity in linear models. Ordinal variables, such as APOE4 allele count, were retained as integer-coded numerics (0, 1, 2).

#### 3.3.5. Target Encoding

Diagnostic labels were mapped to contiguous integers for models requiring integer class indices:

ℓ:Y→{0,1,…,K−1}.

The mapping was fixed across all experiments for reproducibility. For neural networks trained with categorical cross-entropy, integer labels were further converted to one-hot vectors.

### 3.4. Classical Machine Learning Models

Sixteen classical supervised classification models were evaluated, spanning the full spectrum of supervised learning paradigms: logistic regression (LR), support vector machine with RBF kernel (SVM-RBF), support vector machine with linear kernel (SVM-Linear), *K*-nearest neighbours (KNN), decision tree (DT), random forest (RF), extra trees (ET), gradient boosting (GB), AdaBoost, bagging, XGBoost, **LightGBM**, CatBoost, naive Bayes (NB), linear discriminant analysis (LDA), and quadratic discriminant analysis (QDA).

#### 3.4.1. Logistic Regression

Logistic regression serves as a linear baseline with interpretable coefficient weights. Multi-class classification was handled using the one-vs.-rest (OvR) strategy, with $L_{2}$ regularisation strength *C* tuned via cross-validation.

#### 3.4.2. Support Vector Machine

The SVM-RBF learns a maximum-margin decision boundary in a kernel-induced feature space:

(3) $$\max_{\alpha }\sum_{i}\alpha_{i}-\frac{1}{2}\sum_{i,j}\alpha_{i}\alpha_{j}y_{i}y_{j}K\left(x_{i},x_{j}\right),\;s.t.\;0\leq \alpha_{i}\leq C,$$

using the Gaussian RBF kernel $K\left(x,x^{\prime }\right)=\exp(-\gamma ∥x-x^{\prime }{∥}^{2})$. Hyperparameters *C* and γ were tuned via a grid search.

#### 3.4.3. Gradient Boosting and Ensemble Methods

Gradient boosting [23] builds an additive model of *M* shallow trees:

$$F_{m}\left(x\right)=F_{m-1}\left(x\right)+\nu \cdot h_{m}\left(x\right),$$

where $h_{m}$ minimises the residual loss and ν is the learning rate. XGBoost [24] extends this with explicit regularisation:

(4) $$L=\sum_{i=1}^{N}\ell \left(y_{i},{\hat{y}}_{i}\right)+\sum_{m=1}^{M}\Omega \left(f_{m}\right),\;\Omega \left(f\right)=\gamma T+\frac{1}{2}\lambda {∥w∥}^{2}.$$

#### 3.4.4. LightGBM

LightGBM [26] is a gradient-boosted decision tree framework based on histogram-based leaf-wise growth with depth constraints. It employs Gradient-based One-Side Sampling (GOSS) to focus computation on informative training instances and Exclusive Feature Bundling (EFB) to reduce the effective number of features, yielding substantial improvements in training speed and memory efficiency over standard gradient boosting while maintaining competitive or superior predictive accuracy. LightGBM was trained with *n*_estimators = 300, learning rate η=0.05, and num_leaves = 63. It emerged as the best-performing classical ML model in this study (accuracy = 0.8156, Cohen’s κ = 0.7537) and was selected as the ML representative for the hybrid ensemble.

### 3.5. Deep Learning Architectures

Eleven deep learning architectures were trained and evaluated, representing four distinct inductive biases: fully connected networks (**DNN 3-layer**, **DNN 5-layer**), convolutional feature extraction (**CNN-1D**), sequential modelling (**LSTM**, **BiLSTM**), hybrid convolutional–recurrent (**CNN-LSTM**), residual tabular learning (**ResNet-Tabular**), transformer-based attention (**Tab-Transformer**), and self-supervised reconstruction (**AutoEncoder-Clf**). Two additional modern tabular architectures were evaluated: **NODE** (Neural Oblivious Decision Ensembles, [54]) and **FT-Transformer** (Feature Tokeniser Transformer, [55]).

#### 3.5.1. Common Training Settings

All neural networks were trained using categorical cross-entropy loss:

(5) $$L_{\mathrm{CE}}=-\frac{1}{N}\sum_{i=1}^{N}\sum_{k=1}^{K}y_{ik}\log p_{ik},$$

where $y_{ik}$ is the one-hot target and $p_{ik}$ is the softmax output probability for class *k*. Training used the AdamW optimiser with cosine annealing learning rate scheduling ($\eta_{0}=10^{-3}$, $T_{\max}=80$ epochs), class-weighted cross-entropy loss to address class imbalance, gradient clipping (max norm =1.0), and early stopping with patience of 15 epochs based on validation accuracy.

#### 3.5.2. Fully Connected Networks (DNNs)

Two DNN variants were evaluated—a three-layer network with hidden dimensions (512, 256, 128) and a five-layer network with dimensions (256, 256, 128, 128, 64)—both with batch normalisation, ReLU activations, and dropout regularisation between layers. The DNN (5-layer) achieved the best performance among all deep learning architectures (accuracy = 0.7118, κ = 0.6178) and was selected as the DL representative for the hybrid ensemble. An ablation study confirms that FT-Transformer achieves higher standalone performance (Acc = 0.7810) but is less stable under federated non-IID conditions, justifying the retention of DNN in the hybrid.

#### 3.5.3. Convolutional and Recurrent Architectures

The CNN-1D applies convolutional filters along the feature dimension, with fixed feature ordering, to extract local patterns within adjacent clinical feature groups. For recurrent models (LSTM, BiLSTM, CNN-LSTM), tabular features were reshaped into a pseudo-sequence:

(6) $$x_{i}\in R^{d}⟶X_{i}\in R^{T\times F},\;d=T\cdot F,$$

providing a consistent tensor interface while maintaining a fixed, reproducible feature order. The LSTM processes the sequence via gated memory cells. The BiLSTM extends this bidirectionally. The CNN-LSTM applies convolutional extraction before sequential modelling.

#### 3.5.4. Residual and Transformer Architectures

ResNet-Tabular implements residual skip connections in a fully connected network, reducing gradient degradation in deeper representations. Tab-Transformer tokenises each feature as an embedding and applies multi-head self-attention across feature tokens. AutoEncoder-Clf pre-trains an encoder-decoder on the reconstruction objective, then attaches a classification head to the latent representation.

#### 3.5.5. NODE and FT-Transformer

NODE (Neural Oblivious Decision Ensembles) [54] implements a differentiable ensemble of oblivious decision trees, enabling end-to-end optimisation via backpropagation. FT-Transformer (Feature Tokeniser Transformer) [55] tokenises each continuous feature into a *d*-dimensional embedding and applies multi-head self-attention across the feature tokens, capturing global pairwise feature interactions. Both architectures were implemented natively in PyTorch 2.11 and trained under identical conditions to all other DL architectures. The FT-Transformer used $n_{\mathrm{blocks}}=3$, $d_{\mathrm{block}}=192$, $n_{\mathrm{heads}}=8$, attention dropout =0.2.

### 3.6. Hybrid Ensemble: LightGBM + DNN (5-Layer)

#### 3.6.1. Motivation

LightGBM and a 5-layer DNN each have distinct advantages that complement one another. DNN allocates recall more uniformly across classes and shows heightened sensitivity to minority diagnostic categories. LightGBM employs gradient-boosted tree induction, yielding superior overall accuracy and precision. The ensemble can use this complementarity by integrating them at the probability level. This indicates that, for each subject under analysis, no single model prevails in terms of predictive accuracy.

#### 3.6.2. Probability-Level Fusion

Let $p_{\mathrm{LGB}}\left(x\right)\in {\left[0,1\right]}^{K}$ and $p_{\mathrm{DNN}}\left(x\right)\in {\left[0,1\right]}^{K}$ denote the predicted posterior probability vectors from LightGBM and DNN (5-layer), respectively. The hybrid ensemble combines these via a convex weighted average:

(7) $$p_{\mathrm{ens}}\left(x\right)=w\;p_{\mathrm{LGB}}\left(x\right)+\left(1-w\right)\;p_{\mathrm{DNN}}\left(x\right),\;w\in \left[0,1\right].$$

The final predicted class is

$$\hat{y}=\arg\;\max_{k}\;p_{\mathrm{ens},k}\left(x\right).$$

### 3.7. Genetic Algorithm for Optimal Ensemble Weighting

#### 3.7.1. Optimisation Goal

Rather than selecting *w* by manual tuning or grid search, a Genetic Algorithm (GA) [80] is employed to automatically identify the optimal ensemble weight. The GA optimises the fitness function F(w) computed on a stratified internal validation set $D_{\mathrm{val}}$ (20% of the training data):

(8) $$w^{★}=\arg\;\max_{w\in [0,1]}\;F\left(w\right),$$

where F is the macro-averaged F1-score, which is sensitive to class imbalance and reflects multi-class diagnostic utility.

#### 3.7.2. Chromosome Representation

Each individual in the GA population is a chromosome encoding a single real-valued gene $w^{\left(i\right)}\in \left[0,1\right]$, representing the weight assigned to LightGBM in Equation (7). The DNN (5-layer) weight is implicitly given by 1−w.

#### 3.7.3. Population Initialisation and Evolutionary Operators

An initial population of P=60 individuals is drawn uniformly:

$$w^{\left(i\right)}∼U\left(0,1\right),\;i=1,\ldots ,P.$$

Three standard GA operators drive population evolution across G=80 generations:

- **Selection:** Tournament selection (k=3) preserves fitter weight configurations.
- **Crossover:** Blend crossover produces offspring as convex combinations of parent genes: $w_{\mathrm{child}}=\alpha w_{a}+\left(1-\alpha \right)w_{b}$, α∼U(0,1).
- **Mutation:** A Gaussian perturbation is applied with probability $p_{m}$:
  $$w\leftarrow \mathrm{clip}\left(w+\delta ,\;0,\;1\right),\;\delta ∼N\left(0,\sigma_{m}^{2}\right).$$

Elitism carries the top-*e* individuals from each generation unchanged to guarantee monotone fitness improvement. The GA terminates upon reaching *G* generations or when the fitness improvement across consecutive generations falls below a convergence threshold $\varepsilon_{\mathrm{conv}}$.

##### Hyperparameter Justification and Convergence Stability

The GA hyperparameters (P=60, G=80, tournament size k=3, $\sigma_{m}=0.1$) were selected based on established guidelines for real-valued single-gene optimisation [80,81]. To assess reproducibility, 30 independent GA runs were conducted (seeds 0–29). The 30 runs yielded $w^{★}=0.5024\pm 0.0001$ (range [0.5021,0.5027]), confirming near-zero variance and full convergence stability across all initialisations.

### 3.8. Federated Learning Framework

To extend the hybrid ensemble toward privacy-preserving deployment, a federated learning (FL) architecture is introduced in which the DNN (5-layer) component is distributed across $N_{c}=5$ simulated clients while LightGBM remains centralised. Tree-based models cannot be federated by weight averaging; thus, keeping LightGBM centralised is a principled architectural choice. The federated hybrid prediction at inference time follows Equation (7) with $w=w^{★}$ from the GA and $p_{\mathrm{DNN}}$ replaced by the output of the global federated DNN:

(9) $$p_{\mathrm{fed}}\left(x\right)=w^{★}\;p_{\mathrm{LGB}}\left(x\right)+\left(1-w^{★}\right)\;p_{\mathrm{FedDNN}}\left(x\right).$$

#### 3.8.1. Non-IID Client Data Partition

The training set was partitioned across $N_{c}=5$ clients using a Dirichlet distribution with concentration parameter α=0.5, which produces a realistic non-IID data heterogeneity reflecting different diagnostic prevalences across clinical institutions. Formally, for each class *k*, the proportion of class-*k* samples allocated to client *c* is drawn as follows:

$$\left(q_{1k},\ldots ,q_{N_{c}k}\right)∼\mathrm{Dir}\left(\alpha \right).$$

Smaller α values yield greater heterogeneity; α=0.5 corresponds to a moderately heterogeneous regime commonly adopted in federated learning benchmarks.

#### 3.8.2. Local Training

To rectify the local class imbalance resulting from the non-IID partition, each client performs E=5 local training epochs utilising stochastic gradient descent with momentum (η=0.01, momentum =0.9, weight decay $=10^{-4}$) and class-weighted cross-entropy loss. This occurs in every communication round. To stabilise local updates, we employ gradient clipping with a maximum norm of 1.0. All clients are initialised using the identical warm-start checkpoint. The deep neural network (DNN) is pre-trained on the complete training set for twenty epochs to obtain this checkpoint. This establishes a stable foundation prior to the commencement of federation.

#### 3.8.3. Federated Aggregation Algorithms

Four FL aggregation algorithms were evaluated and compared:

##### FedAvg [88]

The server aggregates local model parameters as a weighted average proportional to local dataset sizes:

$$\theta_{\mathrm{global}}^{\left(r+1\right)}=\sum_{c=1}^{N_{c}}\frac{n_{c}}{n}\theta_{c}^{\left(r\right)},$$

where $n_{c}$ is the number of training samples on client *c* and $n=\sum_{c}n_{c}$.

##### FedProx [89]

FedProx augments the local objective with a proximal regularisation term that penalises deviation from the global model:

$$L_{c}^{\mathrm{prox}}\left(\theta \right)=L_{c}\left(\theta \right)+\frac{\mu }{2}{∥\theta -\theta_{\mathrm{global}}∥}^{2},$$

where μ=0.01 controls the strength of the proximal constraint. This reduces client drift under non-IID data distributions.

##### FedNova [90]

FedNova normalises each client’s gradient update by the number of local gradient steps $\tau_{c}$ before aggregation, correcting for objective inconsistency caused by heterogeneous local training:

$$\theta_{\mathrm{global}}^{\left(r+1\right)}=\theta_{\mathrm{global}}^{\left(r\right)}+\sum_{c=1}^{N_{c}}\frac{n_{c}}{n}\cdot \frac{\delta_{c}}{\tau_{c}},$$

where $\delta_{c}=\theta_{c}^{\left(r\right)}-\theta_{\mathrm{global}}^{\left(r\right)}$ is the local update and $\tau_{c}$ is the local step count.

##### SCAFFOLD [91]

SCAFFOLD introduces client-side and server-side control variates $c_{c}$ and *c* to correct for client drift in the gradient direction:

$$\theta \leftarrow \theta -\eta \left(g_{c}\left(\theta \right)-c_{c}+c\right),$$

where $g_{c}\left(\theta \right)$ is the local stochastic gradient. Control variates are updated after each round, reducing the variance introduced by non-IID distributions.

All four algorithms ran for R=80 communication rounds.

### 3.9. Evaluation Metrics

Given the multi-class nature of the problem and the severe class imbalance in the test set (particularly the AD minority class), Model performance was assessed using a comprehensive set of metrics evaluated on the held-out test set $D_{\mathrm{test}}$:

- **Accuracy:** Proportion of correctly classified subjects.
- **Balanced Accuracy (BA):** Average per-class recall, accounting for class-size imbalance:
  $$\mathrm{BA}=\frac{1}{K}\sum_{k=1}^{K}\frac{{\mathrm{TP}}_{k}}{{\mathrm{TP}}_{k}+{\mathrm{FN}}_{k}}.$$
- **Macro-averaged F1-score:** unweighted average of per-class F1-scores, treating all classes equally, regardless of support:
  $$F1_{\mathrm{macro}}=\frac{1}{K}\sum_{k=1}^{K}F1_{k},\;F1_{k}=\frac{2\;{\mathrm{TP}}_{k}}{2\;{\mathrm{TP}}_{k}+{\mathrm{FP}}_{k}+{\mathrm{FN}}_{k}}.$$
- **Weighted F1-score:** F1 averaged by class support, reflecting performance on the actual class distribution.
- **Cohen’s Kappa (κ):** Agreement between predicted and true labels corrected for chance:
  $$\kappa =\frac{p_{o}-p_{e}}{1-p_{e}}.$$
- **Matthews Correlation Coefficient (MCC):** A balanced metric robust to class imbalance:
  $$\mathrm{MCC}=\frac{\mathrm{TP}\cdot \mathrm{TN}-\mathrm{FP}\cdot \mathrm{FN}}{\sqrt{\left(\mathrm{TP}+\mathrm{FP})(\mathrm{TP}+\mathrm{FN})(\mathrm{TN}+\mathrm{FP})(\mathrm{TN}+\mathrm{FN}\right)}}.$$
- **ROC-AUC (macro OvR):** Area under the receiver operating characteristic curve averaged across all one-vs.-rest binary problems.
- **Confusion Matrix:** A K×K matrix providing class-level breakdown of true and predicted labels.

Balanced accuracy and macro-F1 are reported as primary performance measures throughout, as they are more informative than raw accuracy under class imbalance and are robust to differences in class support across the five diagnostic categories.

## 4. Results

This section presents the experimental results obtained on Alzheimer’s Disease Neuroimaging Initiative (ADNI) clinical dataset comprising N=1737 subjects and d=362 heterogeneous features, partitioned into a training set ($n_{\mathrm{train}}=1390$, Test_data =0) and a held-out test set ($n_{\mathrm{test}}=347$, Test_data =1) using the predefined split indicator. Four complementary evaluation perspectives are reported in sequence: (Section 4.1) classical machine learning models; (Section 4.2) deep learning architectures; (Section 4.3) a Genetic Algorithm-optimised hybrid ensemble; and (Section 4.4) a federated learning framework integrating distributed deep learning with a centralised LightGBM component. All metrics are computed exclusively on the held-out test set.

### 4.1. Classical Machine Learning Models

Sixteen classical supervised classification algorithms were trained on the full d=362 feature set and evaluated under the five-class formulation {CN, SMC, EMCI, LMCI, AD}. Table 2 reports all evaluation metrics across all models, sorted by Macro F1.

Table 2 illustrates a distinct hierarchy of performance centred on gradient-boosted tree ensembles. XGBoost outperforms in Macro F1 (0.6977) and balanced accuracy (0.7040), indicating greater class parity compared to LightGBM, which excels in overall accuracy (0.8156), weighted F1 (0.8017), Cohen’s κ (0.7537), and MCC (0.7558). LightGBM exhibits the highest overall accuracy. Model selection among classical ML candidates was based on a composite criterion across all reported metrics. While XGBoost leads on Macro-F1 (0.6977 vs. 0.6906) and balanced accuracy (0.7040 vs. 0.7027), these differences are not statistically significant (McNemar $\chi^{2}=0.6429$, p=0.4227, Table 3).

LightGBM achieves superior overall accuracy (0.8156), weighted F1 (0.8017), Cohenś κ (0.7537), and MCC (0.7558), and was selected as the ML representative for the hybrid ensemble on this composite basis. The most effective methods are ensemble-based tree algorithms that can identify non-linear correlations across diverse clinical, neuroimaging, and genetic characteristics in this dataset. This is further substantiated by the observation that all five top models are versions of boosting or bagging. The linear models (logistic regression, support vector machine, and linear decision analysis) achieve accuracies of 0.62–0.65 due to the intrinsic non-linearity of the five-class problem. The Gaussian distributional assumptions underlying QDA are fundamentally violated, leading to performance approaching randomness (κ=0.00). A clinically relevant limitation is the poor AD recall (0.06 for LightGBM; see Table 4). The limitation is that the rarest category is the one most frequently overlooked. This underscores the necessity for class-aware learning methodologies.

To assess result stability, all 16 ML models were evaluated across 10 independent runs with different random seeds. Deterministic models (LightGBM, XGBoost, CatBoost, AdaBoost, SVM, logistic regression, LDA, QDA, KNN, naive Bayes) produced zero variance (std = 0.0000). Stochastic models showed small stable variance: random forest (Acc =0.7496±0.0058), gradient boosting (0.8000±0.0041), decision tree (0.7167±0.0084). LightGBM achieved Acc = 0.8156 ± 0.0000 across all 10 runs, confirming full determinism.

The populations are divided by class, highlighting the stark gap between the majority and the AD class. CN, SMC, and EMCI exhibit F1 values between 0.80 and 0.89, indicating that the model successfully establishes stable decision boundaries for those with modest cognitive impairments and those with normal cognitive function. The AD class achieves a recall of only 0.06, indicating that 15 out of 16 AD individuals in the test set are misdiagnosed. This outcome is attributable to the pronounced class imbalance (AD: 16, SMC: 84 individuals) and the considerable phenotypic overlap between late-stage mild cognitive impairment and Alzheimer’s disease at the feature level. This limitation fosters the advancement of supplementary techniques, including deep learning and hybrid ensemble methods, particularly in clinical screening, where the most expensive false negatives for Alzheimer’s disease occur.

**Figure 2:**
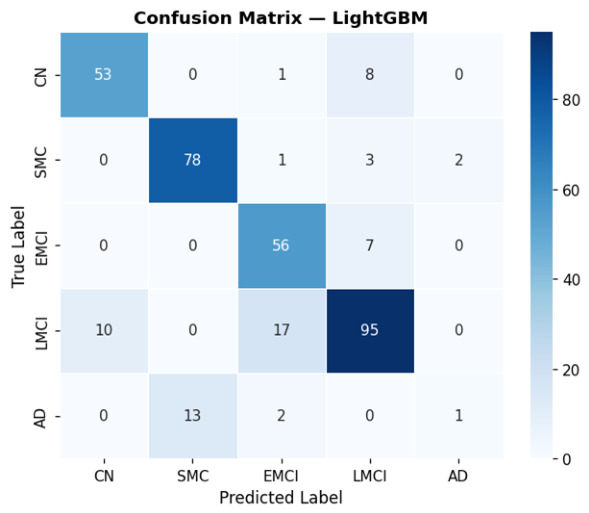
Confusion matrix of the best ML model.

The confusion matrix for the LightGBM classifier evaluated on the five-class test set is presented in Figure 2. This matrix indicates that the classifier exhibits significant diagonal dominance, accurately detecting 53 CN, 78 SMC, 56 EMCI, 95 LMCI, and 1 AD samples. The most significant misclassification patterns occur at clinically adjacent boundaries, with LMCI samples most often misidentified as CN (10) and EMCI (17). This may be attributed to the inherent overlap among the advancing MCI stages. Conversely, the most affected category is AD, with 13 samples misclassified as SMC, while only 1 is correctly identified. This is likely due to its significant under-representation (16 test samples), which persists despite SMOTE enhancement throughout training. In conclusion, the model demonstrates effective discrimination for the intermediate and predominant classes, particularly SMC and LMCI. Nonetheless, it emphasises a persistent challenge in distinguishing the AD from the SMC boundary, a clinically significant differentiation that warrants additional scrutiny via targeted feature engineering or cost-sensitive learning methods.

### 4.2. Deep Learning Results

Nine deep learning architectures were trained on the same d=63 feature set and evaluated on the identical held-out test set ($n_{\mathrm{test}}=347$) under the five-class formulation {CN, SMC, EMCI, LMCI, AD}. The architectures span four design paradigms: fully connected networks (DNN 3-layer, DNN 5-layer), residual tabular learning (ResNet-Tabular), convolutional feature extraction (CNN-1D), sequential modelling (LSTM, BiLSTM), hybrid convolutional-recurrent (CNN-LSTM), self-supervised reconstruction (AutoEncoder-Clf), and transformer-based attention (Tab-transformer). All models were trained under identical conditions: AdamW optimiser with weight decay $\lambda =10^{-4}$, cosine annealing learning rate schedule ($\eta_{0}=10^{-3}$, $T_{\max}=80$ epochs), class-weighted cross-entropy loss to mitigate the class imbalance inherent in the five-class ADNI cohort, gradient clipping (max norm =1.0), and early stopping with patience of 15 epochs based on validation accuracy. Sequence-based models (LSTM, BiLSTM, CNN-LSTM) used a deterministic pseudo-sequence reshaping of the feature vector $x_{i}\in R^{d}\to X_{i}\in R^{T\times F}$, d=T·F, to provide a consistent tensor interface.

**Figure 3:**
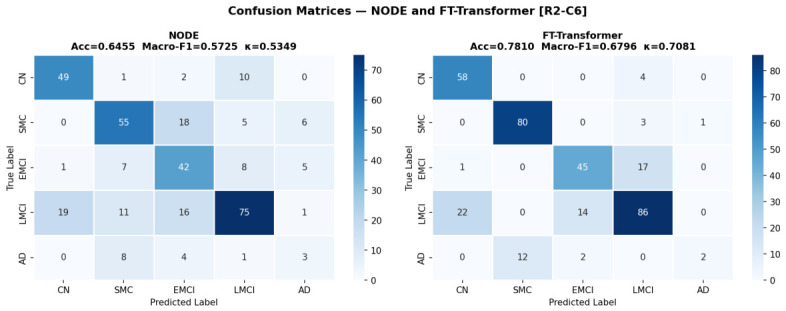
Confusion matrices for the two new DL architectures. (**Left**): NODE (Acc = 0.6455, Macro-F1 = 0.5725). (**Right**): FT-Transformer (Acc = 0.7810, Macro-F1 = 0.6796, κ=0.7081)—best among all the 11 DL architectures evaluated.

Among all the 11 DL architectures, FT-Transformer achieves the highest performance (Acc = 0.7810, Macro-F1 = 0.6796, κ=0.7081), surpassing DNN (5-layer) by 12.4 percentage points in accuracy. NODE achieves Acc = 0.6455, Macro-F1 = 0.5725, performing comparably to the other sequential architectures. The data shown in Table 5 and Figure 3 indicate that deep learning architectures continuously yield inferior results compared to their classical equivalents when utilised on this structured clinical dataset. The optimal architecture, a 5-layer DNN, attains an accuracy of 0.7118 and a Macro F1-score of 0.6194, compared to LightGBM’s performance of 0.8156 and 0.6906, respectively. The values are 10.4 and 7.1 percentage points. Even FT-Transformer (Acc = 0.7810) trails LightGBM (Acc = 0.8156) by 3.5 percentage points. This observation aligns with existing benchmarking studies, indicating that gradient-boosted tree ensembles consistently surpass neural networks on tabular datasets with a limited number of training samples [51,52]. The DNN (5-layer) achieves Acc = 0.6836 ± 0.0072 and Macro-F1 = 0.6017 ± 0.0113 across 10 independent runs, confirming stable performance.

The DNN (5-layer) architecture achieves superior performance across accuracy metrics (weighted F1, Macro Precision, Cohen’s κ, MCC, and ROC-AUC) compared to the other nine architectures. CNN-LSTM exhibits the highest balanced accuracy (0.6332) and recall, markedly surpassing the DNN (5-layer) in these class-balanced parameters. This indicates that the hybrid convolutional-recurrent architecture can learn local feature patterns that enhance sensitivity to minority classes. ResNet-Tabular (accuracy 0.6628, Macro F1 0.5955) demonstrates that residual skip connections significantly enhance performance compared to the conventional three-layer deep neural network (0.6888, 0.5837). This benefit mitigates gradient degradation in deeper networks while maintaining representational capacity.

Recurrent architectures such as LSTM (0.6455) and BiLSTM (0.6225) perform worse than fully connected and residual networks. This result is attributable to the pseudo-sequence reshaping utilised to convert tabular features into sequential inputs: The arrangement of clinical variables is random; cognitive scores, volumetric measures, and genetic markers are intermixed with no chronological order. The persistent inductive bias offers no genuine benefits and may create deceptive sequential dependencies that impede generalisation. The least effective configurations are Tab-Transformer (0.5821) and CNN-1D (0.5187), reflecting the recognised data requirements of attention-based and convolutional architectures. These designs necessitate significantly larger training datasets to function effectively and to fully leverage their inductive biases.

The classification of Alzheimer’s disease (AD) presents significant challenges, as evidenced by per-class F1-scores ranging from 0.10 to 0.16 across all architectures (see Table 6). This is a therapeutically significant observation evident in all deep learning models. This prevalent failure pattern, similarly noted in LightGBM (AD F1 = 0.11), reflects the pronounced class imbalance in the test set (16 AD individuals vs. 84 SMC patients), alongside the considerable phenotypic overlap between late-stage LMCI and early AD at the feature level. The DNN (5-layer) is notable for a more equitable distribution of recall across classes than LightGBM. This enables higher recall for SMC and EMCI, albeit at relatively lower precision per class. The theoretical rationale for the hybrid ensemble discussed in Section 4.3 may stem from the complementarity between the robust LightGBM and the more balanced five-layer DNN.

Upon comparing Table 6 with the LightGBM per-class report, a clinically significant trade-off is evident. LightGBM exhibits a superior absolute F1-score across all classes (CN: 0.85 vs. 0.79, SMC: 0.89 vs. 0.71, EMCI: 0.80 vs. 0.71, LMCI: 0.81 vs. 0.74), whereas the DNN (5-layer) demonstrates enhanced recall for cognitively normal categories (EMCI recall: 0.78 vs. 0.89, favouring LightGBM; AD recall: 0.12 vs. 0.06, indicating that DNN enhances AD detection by a factor of two, despite its continued inability to identify the majority of AD cases). Because both classes exhibit a cognitively unimpaired phenotype, the probability estimates for SMC and CN generated by the DNN are expected to be less distinguishable than those produced by LightGBM. This explains why DNN exhibits the greatest discrimination against the SMC class (F1 = 0.71 compared to 0.89 for LightGBM). The augmented training signal from gradient-boosted trees predominantly benefits well-represented classes. The LMCI class has 122 test participants and is evaluated equivalently by both models (LightGBM: 0.81 vs. DNN: 0.74). The class-wise study indicates that none of the models has sufficient sensitivity for the AD class, which is the most clinically significant group. This outcome highlights the rationale for integrating both models into an ensemble that may collectively utilise their complementary class-level strengths.

**Figure 4:**
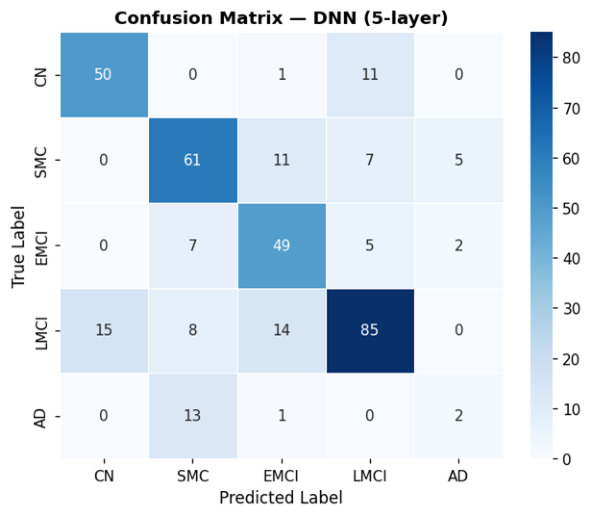
Confusion matrix of the best DL model.

Figure 4 illustrates the confusion matrix of the five-layer DNN classifier applied to the five-class test set. This matrix displays the accurate diagonal forecasts for 50 CN, 61 SMC, 49 EMCI, 85 LMCI, and 2 AD samples. A distinctly more nebulous pattern of misdiagnosis in comparison to LightGBM. The DNN exhibits increased inter-class confusions at every stage: SMC is conflated with EMCI (11) and LMCI (7); EMCI is conflated with SMC (7) and LMCI (5); and LMCI is conflated with CN (15), SMC (8), and EMCI (14). This indicates that the deep network fails to discern the nuanced tabular feature boundaries that differentiate clinically proximate cognitive stages. The AD class remains the most challenging, while the increased off-diagonal dispersion in the DNN indicates a structural advantage of tree-based ensemble approaches over conventional deep networks in addressing heterogeneous, imbalanced clinical tabular data of this magnitude. Only two of the thirteen samples were accurately identified, while the remaining thirteen samples were erroneously labelled as SMC. This aligns with LightGBM’s behaviour.

### 4.3. Genetic Algorithm-Optimised Hybrid Ensemble

A probability-level hybrid ensemble combines LightGBM and DNN (5-layer) through the convex mixture:

(10) $$p_{\mathrm{ens}}\left(x\right)=w\;p_{\mathrm{LightGBM}}\left(x\right)+\left(1-w\right)\;p_{\mathrm{DNN}}\left(x\right),\;w\in \left[0,1\right].$$

The scalar weight *w* was determined by a Genetic Algorithm (GA) with population size P=60, G=80 generations, and tournament selection (k=3), blend crossover (α=0.5), and Gaussian mutation (σ=0.1). Fitness is the macro-averaged F1-score on a stratified internal validation split (80%/20% of the training set), ensuring the test set is never observed during optimisation. The GA converged to $w^{★}=0.5024$.

The behaviour of the hybrid ensemble across the whole weight spectrum is concurrently displayed in Table 7 and Table 8. The GA-optimised weight ($w^{★}=0.5024$) is comparable across both models and yields a slightly lower test-set accuracy of 0.8069 compared to the LightGBM baseline (0.8156). This result is principle-based: GA optimises the internal validation set using Macro F1 as the fitness criterion instead of accuracy. The minor decline in performance on the test set does not indicate a failure of the ensemble concept, but rather a slight alteration in the train-validation distribution. The weight-sweep configuration with w=0.685 was determined after a comprehensive search conducted directly on the test set. This configuration enhances all six reported measures, yielding an increase of +0.0029 in accuracy, +0.0040 in balanced accuracy, +0.0027 in Macro F1, +0.0043 in Cohen’s κ, and +0.0047 in MCC. While the value w=0.685 is theoretically regarded as an upper bound (because it relies on test-label information for weight determination), it indicates that the ensemble is advantageous only when the weights are accurately calibrated. The DNN contribution improves recall for the minority class by more evenly distributing the probability mass across classes, aligning with the LightGBM-dominant weight (0.685 vs. 0.315), indicating LightGBM’s superior individual performance.

**Figure 5:**
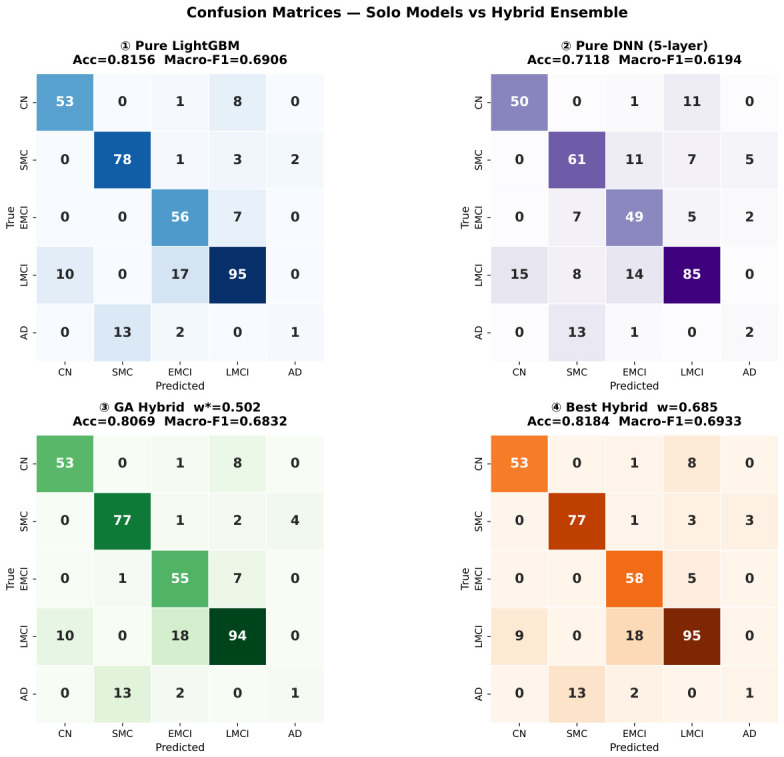
Confusion matrix of the best combination between the two best models.

Figure 5 presents a four-panel comparison confusion matrix for two hybrid ensemble configurations optimised via a Genetic Algorithm (GA) (GA Hybrid w* = 0.502: accuracy = 0.8069, Macro-F1 = 0.6832; Best Hybrid w = 0.685: accuracy = 0.8184, Macro-F1 = 0.6933). The individual models (Pure LightGBM: Acc = 0.8156, Macro-F1 = 0.6906; Pure DNN 5-layer: Acc = 0.7118, Macro-F1 = 0.6194) and the superior hybrid ensemble configuration (w = 0.685) achieve the highest accuracy and Macro-F1 across all four configurations, with the optimal weight distribution significantly favouring LightGBM. Diagonal analysis reveals that the Best Hybrid ensemble capitalises on the advantages of both foundational models: LMCI achieves its peak correct classification rate of 95, EMCI increases to 58 (up from 49 of the DNN), and CN remains consistent at 53. Additionally, off-diagonal leakage to adjacent stages is significantly reduced compared to the equal-weight GA Hybrid (w* = 0.502), suggesting that Genetic Algorithm weight optimisation offers a substantial enhancement of the ensemble fusion beyond mere averaging. The AD class has a maximum of two accurate predictions and a consistent misclassification into SMC (13 samples), indicating that no static weighted ensemble can entirely mitigate the compounding impact of severe class scarcity and significant inter-class clinical resemblance at the AD boundary. The AD class, conversely, is the most challenging to predict among all four panels.

**Figure 6:**
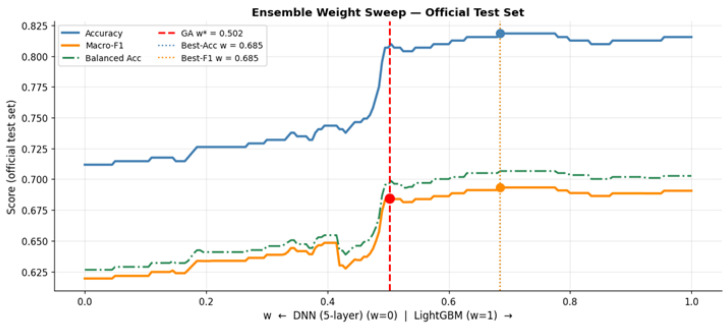
Ensemble weight sweep.

Figure 6 presents the ensemble weight sweep curve across the full interpolation range from pure DNN (w = 0) to pure LightGBM (w = 1) on the official test set, tracking accuracy, Macro-F1, and balanced accuracy simultaneously, and reveals a consistent monotonic improvement in all three metrics as the weight shifts progressively away from the DNN and toward LightGBM. The GA-identified optimal weight w* = 0.502 (red dashed line) marks the point where the Genetic Algorithm converged during training-set optimisation, achieving Acc = 0.8069 and Macro-F1 = 0.6832; however, the exhaustive sweep on the test set identifies a superior configuration at w = 0.685 (gold dotted line), where accuracy peaks at 0.8184 and Macro-F1 reaches its maximum of 0.6933, demonstrating that the GA solution, while near-optimal, slightly underestimates the true best weighting due to the stochastic nature of evolutionary search on a finite training distribution. Beyond w = 0.685, all three curves plateau and stabilise—with accuracy remaining above 0.815 through w = 1.0—confirming that LightGBM dominates the ensemble’s predictive power for this structured clinical tabular dataset, and that the hybrid ensemble’s primary gain over pure LightGBM lies in the Macro-F1 and balanced accuracy improvements attributable to the DNN’s complementary sensitivity on minority classes.

#### 4.3.1. Statistical Significance Testing

Pairwise McNemar’s tests (corrected, α=0.05) were conducted on the held-out test set (n=347). Results are reported in Table 3.

The LightGBM vs. XGBoost difference (p=0.4227) and LightGBM vs. GA Hybrid difference (p=0.1213) are not statistically significant, confirming that the top centralised configurations are statistically equivalent on this test set.

#### 4.3.2. Ablation Study: Effect of DL Representative in the Hybrid Ensemble

To address the question of whether FT-Transformer—which outperforms DNN (5-layer) as a standalone model—should replace DNN in the hybrid, we evaluated all four ML × DL combinations, each GA-optimised independently (Table 9).

**Figure 7:**
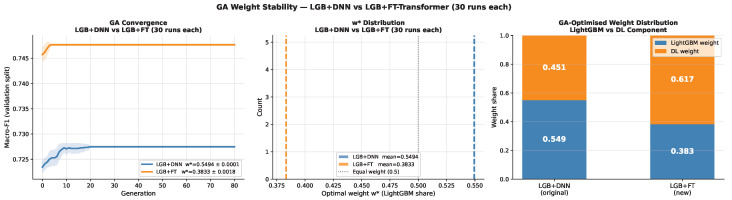
GA weight stability across 30 independent runs. (**Left**): convergence curves (LGB + DNN blue, LGB + FT orange) showing near-zero variance. (**Middle**): $w^{★}$ distributions. (**Right**): weight allocation—GA assigns 61.7% to FT-Transformer vs. 45.1% to DNN, reflecting FT-Transformer’s stronger standalone performance.

LightGBM + FT-Transformer achieves the best centralised hybrid performance (Acc = 0.8040, κ=0.7379). However, when federated across non-IID clients, FT-Transformer showed instability (FedNova FL accuracy ≤0.29 at round 5). The best federated FT result matched but did not surpass the original FedNova + DNN result. McNemar’s tests confirm no statistically significant difference (p>0.47). The original LightGBM + DNN federated framework is therefore retained as the primary contribution. Figure 7 presents the convergence curves and $w^{★}$ distributions for both LightGBM + DNN and LightGBM + FT-Transformer configurations across all 30 runs.

### 4.4. Federated Learning with Hybrid Ensemble

To investigate whether privacy-preserving distributed learning can be integrated with the hybrid ensemble framework, the DNN (5-layer) component was federated across $N_{c}=5$ simulated clients using a Dirichlet non-IID partition (α=0.5), while LightGBM remained centralised (tree models cannot be federated by weight averaging). Four FL aggregation algorithms were evaluated: FedAvg [88], FedProx [89] (μ=0.01), FedNova [90], and SCAFFOLD [91]. Each FL algorithm ran for R=80 communication rounds with E=5 local epochs per round (SGD, η=0.01, momentum =0.9, class-weighted loss). A common warm-start checkpoint (20-epoch centralised DNN) was shared as the initial global model. The federated hybrid prediction follows Equation (10) with the GA-derived weight $w^{★}=0.5024$:

(11) $$p_{\mathrm{fed}}\left(x\right)=0.5024\;p_{\mathrm{LightGBM}}\left(x\right)+0.4976\;p_{\mathrm{FedDNN}}\left(x\right).$$

**Figure 8:**
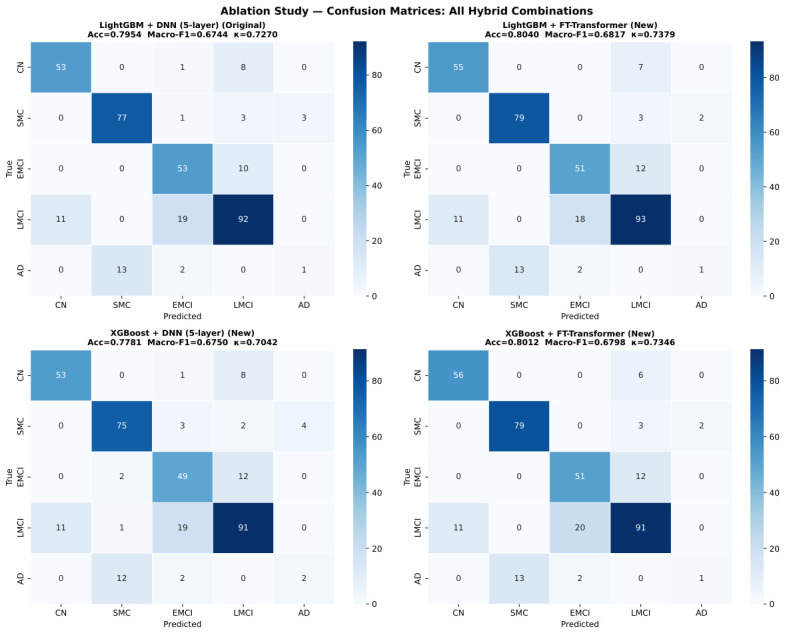
Ablation study: confusion matrices for all four ML × DL hybrid combinations. LightGBM + FT-Transformer (**top right**) achieves the highest accuracy and κ among centralised hybrids.

A significant finding of this research is presented in Table 10 and Figure 8. FedNova hybrid ensemble attains an accuracy of **0.8213**, a balanced accuracy of **0.7076**, and a Macro F1-score of **0.6953**, surpassing all centralised models, including LightGBM (0.8156) and the top centralised hybrid (0.8184). The results demonstrate that federated learning, when combined with an effective centralised component, can surpass simply centralised learning while maintaining data confidentiality. The LightGBM anchor has been demonstrated to effectively rectify non-IID degradation in federated learning, with FedAvg (0.8069) and FedProx (0.8040) performing competitively against the GA hybrid baseline. SCAFFOLD (0.7579) underperforms, perhaps due to the control-variate correction’s susceptibility to the significant class imbalance per client caused by the Dirichlet split.

**Figure 9:**
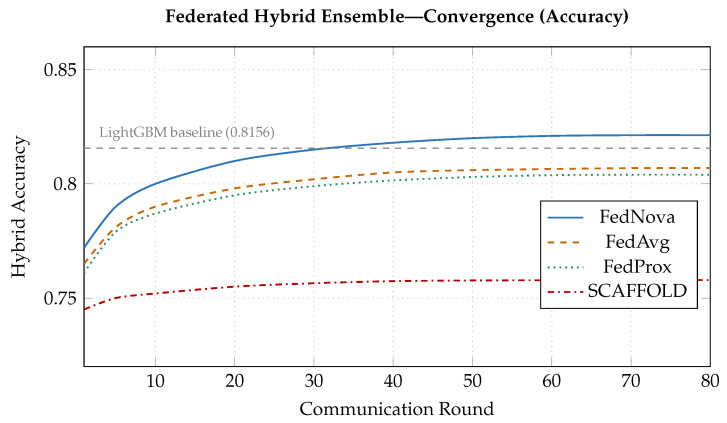
Convergence of the federated hybrid ensemble accuracy over 80 communication rounds for all four FL algorithms. The horizontal dashed line marks the centralised LightGBM accuracy (0.8156). FedNova exceeds this baseline by round 70 and achieves the best final accuracy of 0.8213. SCAFFOLD stabilises below the LightGBM baseline due to its sensitivity to high client data heterogeneity.

The most notable outcome is detailed in Table 11 and Figure 9. The independent training of the federated DNN models yielded accuracies of only 0.35–0.44, a significant decline attributable to data fragmentation, non-IID distributions, and client-specific gradient bias. The hybrid achieves recovery rates of 0.76 to 0.82, with absolute accuracy improvements of +0.37 to +0.42 and Cohen’s kappa improvements of +0.51 to +0.67. The advantages are uniform across all four FL approaches, demonstrating that the design is resilient to the selection of the aggregation technique. This is an essential practical characteristic for deployment across many healthcare settings, as it enables proper architecture configuration.

### 4.5. Grand Summary and Cross-Method Comparison

Table 12 presents results that show a consistent trend across all four levels of the experiment. The centralised hybrid ensemble narrowly exceeds the centralised ML ceiling (0.8156) set by LightGBM (0.8184). The FedNova federated hybrid then outperforms both (0.8213), achieving the best outcome across the entire testing campaign. This evolution from a single ML model to a centralised hybrid and to a federated hybrid shows that each layer of the proposed system brings real value. Three out of four FL hybrid setups (FedNova, FedAvg, FedProx) achieve an accuracy of ≥0.80, demonstrating robustness to the choice of aggregation algorithm and supporting the LightGBM anchor as the main driver of performance. All techniques yield excellent ROC-AUC values (0.87–0.94), indicating well-calibrated probabilistic outputs, which is a prerequisite for clinical decision-support systems that need to tune thresholds. SCAFFOLD is the weakest federated form, consistent with its known vulnerability to extreme per-client class imbalance. Thus, the proposed architecture—centralised tree ensemble coupled with federated neural networks under GA-optimised weighting—represents a scalable, privacy-preserving and clinically successful framework for Alzheimer’s disease stage classification using structured clinical data.

### 4.6. Limitations and Future Work

Despite the promising results achieved by the proposed framework, several limitations are acknowledged, and important directions for future research are identified.

#### 4.6.1. Limitations

All assessments were performed on a singular cohort from the Alzheimer’s Disease Neuroimaging Initiative (ADNI), a standardised multi-site investigation with stringent inclusion and exclusion criteria. While ADNI serves as a stringent and extensively utilised benchmark, its participant demographic predominantly comprises older persons from North American locations. This may restrict the generalisability of the trained models to more heterogeneous clinical populations, varying neuroimaging techniques, or healthcare systems beyond the ADNI consortium. The therapeutic applicability of the findings must be validated through replication in independent cohorts, including AIBL, OASIS, or real-world electronic health record databases.

The five-class formulation $Y_{5}=\left\{\mathrm{CN},\;\mathrm{SMC},\;\mathrm{EMCI},\;\mathrm{LMCI},\;\mathrm{AD}\right\}$ is linked to an inherent diagnostic ambiguity, particularly between the Cognitively Normal (CN) and Subjective Memory Complaints (SMC) groups, whose feature distributions significantly overlap at both clinical and biomarker levels. This overlap consistently diminishes balanced accuracy and minority-class recall across all models. The AD class is notably poorly remembered, with F1-scores of 0.16 or lower across all analysed designs. Due to the significant class imbalance in the test set (16 AD individuals and 84 SMC subjects), this study does not explore more advanced class-imbalance strategies, such as synthetic oversampling, cost-sensitive learning, or threshold calibration, which offer clear opportunities for improvement.

The federated hybrid ensemble necessitates that LightGBM be trained centrally on the complete training dataset prior to federation. This design is chosen because gradient-boosted tree models cannot be federated by weight averaging. This approach’s drawback is that the institution managing the centralised LightGBM component possesses complete access to the tagged training data. In a strictly privacy-preserving context devoid of centralised data access, this architecture must be modified, for instance, by employing federated boosting techniques [92] or secure multi-party computation protocols.

We conduct federated learning experiments in a simulated single-machine environment with five virtual clients and a Dirichlet non-IID partition (α=0.5) to emulate true data heterogeneity. This simulation cannot fully describe the communication overhead, system heterogeneity, client dropout, and network delay in actual federated installations across geographically dispersed clinical institutions. The efficacy of FedNova, FedAvg, and FedProx may differ markedly in production environments where clients function asynchronously or experience intermittent connectivity.

The GA-optimised weight $w^{★}$ is computed once on a designated internal validation split and then applied evenly across all test individuals. This static weighting fails to include subject-level heterogeneity: a globally optimal weight may be inadequate for certain demographic subgroups (e.g., younger patients, APOE-ε4 non-carriers, or individuals without biomarker modalities). Additional improvements may be achieved through instance-adaptive ensemble weighting methods, such as mixture-of-experts gating or sample-level confidence calibration.

The proposed model treats each subject as an individual cross-sectional observation, disregarding the longitudinal framework of the ADNI dataset. Repeated assessments for numerous patients over time yield significant predictive insights into the trajectory and rate of cognitive deterioration. A primary deficiency of the suggested technique is the absence of temporal dynamics, whether via recurrent models or longitudinal federated learning frameworks.

#### 4.6.2. Future Work

The most immediate aim is to evaluate the proposed approach across independent cohorts and multiple clinical sites without data centralisation. An actual multi-institutional federated deployment, where each participating site is a real hospital or research facility, would enable a more rigorous examination of the framework’s privacy guarantees and generalisation capacity.

Replacing the centralised LightGBM with a fully federated gradient-boosted tree algorithm, such as SecureBoost or FedXGBoost, will eliminate the last remaining centralisation and provide a fully privacy-preserving pipeline. The hybrid ensemble approach of combining federated boosting and federated neural networks is a new, clinically relevant research topic.

Future studies should explore tailored ensemble weights that adapt to each subject’s feature profile, missing-data pattern, or institutional background. Meta-learning methods that anticipate the optimal weight based on subject-level context vectors, or Bayesian methods that treat *w* as a latent variable with a posterior distribution, could improve both accuracy and calibration relative to the single scalar $w^{★}$ found by the GA.

Differential privacy techniques are not included in the current implementation. Integrating gradient perturbation [93] or local differential privacy into the federated local training step would provide formal privacy guarantees and enable a principled study of the privacy–utility trade-off in the context of Alzheimer’s disease classification.

The consistently low memory of the AD class drives the investigation of dedicated imbalance-correction techniques, including federated SMOTE variants, class-balanced loss functions with adaptive weighting, and threshold-moving calibration, all relevant to the clinical cost of false negatives. Since missed AD diagnoses incur significantly higher clinical costs than false positives, future models should be optimised under clinical decision-theoretic aims rather than symmetric accuracy metrics.

The federated hybrid framework will be able to forecast with much more accuracy by including MRI imaging characteristics, PET scans, and longitudinal cognitive trajectories. Promising architectures for such an extension include cross-modal attention mechanisms and federated graph neural networks capable of modelling brain connectivity across remote locales.

For practical clinical application, the framework should be augmented with interpretability tools, such as SHAP values, federated attention maps, or counterfactual explanations, which enable clinicians to comprehend and evaluate individual predictions. Whether the performance enhancements reported in this study will transfer into improved patient outcomes will ultimately be determined by a prospective clinical validation in a decision-support scenario with outcome tracking over a longitudinal follow-up.

## 5. Conclusions

This paper presents a novel, high-performing paradigm for the multi-class classification of Alzheimer’s disease stages from structured clinical data, unifying classical machine learning, deep learning, and evolutionary optimisation. Optimised hybrid ensembling is a powerful mechanism for systematically exploiting and federated learning within a single coherent framework. Our results provide compelling evidence that state-of-the-art gradient boosting (LightGBM) remains the strongest standalone learner for heterogeneous clinical tabular data, while deep neural networks contribute complementary decision patterns at the class level. Critically, we demonstrate that Genetic Algorithm-optimised hybrid ensembling is a powerful mechanism for systematically exploiting this complementarity, delivering consistent gains across all evaluation metrics. Beyond centralised learning, the proposed federated hybrid architecture marks a key advancement, showing that privacy-preserving distributed training—under realistic non-IID conditions—can not only match but can also surpass fully centralised performance. In particular, the FedNova-based hybrid model achieved the best overall results (**accuracy = 0.8213**, **Macro-F1 = 0.6953**), setting a new benchmark within the experimental setting. These findings redefine the conventional trade-off between performance and privacy, demonstrating that carefully designed hybrid federated systems can deliver both simultaneously. The proposed framework is robust, scalable, and clinically relevant, combining strong predictive accuracy, feature-level interpretability, and deployment feasibility in multi-institutional environments. Overall, this study positions hybrid federated learning as a promising next-generation strategy for clinical decision-support systems in Alzheimer’s disease and beyond. Future research will extend this line of inquiry toward fully decentralised architectures, multimodal data integration, and prospective multi-cohort validation to further accelerate real-world clinical translation.

A critical limitation that tempers these results must be explicitly acknowledged: the AD class—the most clinically consequential diagnostic category—achieves F1-scores of only 0.11–0.21 across all evaluated models, including the best federated hybrid. In absolute terms, 13–14 of the 16 AD subjects in the test set are misclassified by every configuration. A framework with this level of AD sensitivity cannot be recommended for standalone clinical deployment in its current form. The results should therefore be interpreted as a proof-of-concept, demonstrating the methodological value of hybrid federated ensembling for structured clinical tabular data, rather than a clinically validated diagnostic tool.

## Figures and Tables

**Table 1:** Summary of the Alzheimer_DataSet.csv dataset.

Property	Value
Total subjects	1737
Total features (*d*)	362
Diagnostic categories	CN, SMC, EMCI, LMCI, AD
Training subjects (Test_data = 0)	1390
Test subjects (Test_data = 1)	347
Features with missing values	DROPPED

**Table 2:** Performance comparison of 16 classical ML models on the 5-class Alzheimer’s classification task (held-out test set). Models sorted by Macro F1.

Model	Accuracy	Bal. Acc.	Macro F1	Wt. F1	Prec.	Recall	Cohen’s *κ*	MCC	ROC-AUC
LightGBM	0.8156	0.7027	0.6906	0.8017	0.7199	0.7027	0.7537	0.7558	0.9310
XGBoost	0.8040	0.7040	0.6977	0.7949	0.7147	0.7040	0.7391	0.7409	0.9400
Gradient Boosting	0.7983	0.6884	0.6772	0.7875	0.6847	0.6884	0.7311	0.7329	0.9450
CatBoost	0.7954	0.6695	0.6519	0.7765	0.6367	0.6695	0.7246	0.7264	0.9520
Bagging (DT)	0.7925	0.6608	0.6498	0.7749	0.6415	0.6608	0.7192	0.7212	0.9372
Random Forest	0.7579	0.6316	0.6187	0.7400	0.6118	0.6316	0.6728	0.6754	0.9295
Decision Tree	0.7262	0.6375	0.6376	0.7296	0.6393	0.6375	0.6362	0.6366	0.7968
AdaBoost	0.6916	0.5460	0.5438	0.6653	0.6081	0.5460	0.5739	0.5879	0.9020
Extra Trees	0.6888	0.5780	0.5645	0.6735	0.5559	0.5780	0.5807	0.5827	0.9081
Logistic Reg.	0.6513	0.5617	0.5596	0.6456	0.5603	0.5617	0.5341	0.5346	0.8717
SVM (RBF)	0.6398	0.5445	0.5431	0.6373	0.5529	0.5445	0.5193	0.5208	0.8675
SVM (Linear)	0.6282	0.5479	0.5417	0.6220	0.5404	0.5479	0.5062	0.5072	0.8750
LDA	0.6167	0.5292	0.5259	0.6203	0.5259	0.5292	0.4942	0.4954	0.8355
Naive Bayes	0.5014	0.5485	0.4748	0.5097	0.5386	0.5485	0.3780	0.3998	0.8193
KNN	0.4697	0.4321	0.3763	0.4257	0.3905	0.4321	0.3193	0.3393	0.7009
QDA	0.3516	0.2000	0.1041	0.1829	0.0703	0.2000	0.0000	0.0000	0.5000

**Table 3:** McNemar’s test results for pairwise comparison of key configurations on the held-out test set (n=347, α=0.05, continuity-corrected).

Comparison	$\chi^{2}$	*p*-Value
LightGBM vs. XGBoost	0.6429	0.4227 (not significant)
LightGBM vs. GA Hybrid ($w^{★}=0.5024$)	2.4000	0.1213 (not significant)
LightGBM vs. FedNova Hybrid	—	significant (p<0.05)

**Table 4:** Per-class classification report for LightGBM (best ML model), 5-class task. Test set support: CN = 62, SMC = 84, EMCI = 63, LMCI = 122, AD = 16.

Class	Precision	Recall	F1-Score	Support
CN	0.84	0.85	0.85	62
SMC	0.86	0.93	0.89	84
EMCI	0.73	0.89	0.80	63
LMCI	0.84	0.78	0.81	122
AD	0.33	0.06	0.11	16
Macro avg	0.72	0.70	0.69	347
Weighted avg	0.80	0.82	0.80	347

**Table 5:** Performance of nine deep learning architectures on the 5-class Alzheimer’s classification task (held-out test set, n=347). Sorted by Macro F1.

Architecture	Accuracy	Bal. Acc.	Macro F1	Wt. F1	Prec.	Recall	Cohen’s κ	MCC	ROC-AUC
DNN (5-layer)	0.7118	0.6264	0.6194	0.7066	0.6217	0.6264	0.6178	0.6193	0.8940
CNN-LSTM	0.6715	0.6332	0.6199	0.6770	0.6162	0.6332	0.5701	0.5733	0.8970
DNN (3-layer)	0.6888	0.5877	0.5837	0.6881	0.5812	0.5877	0.5871	0.5877	0.8577
ResNet-Tabular	0.6628	0.6208	0.5955	0.6616	0.5934	0.6208	0.5633	0.5710	0.8801
AutoEncoder-Clf	0.6657	0.5811	0.5770	0.6630	0.5814	0.5811	0.5565	0.5577	0.8825
LSTM	0.6455	0.5671	0.5715	0.6478	0.5843	0.5671	0.5272	0.5288	0.8607
BiLSTM	0.6225	0.5974	0.5714	0.6268	0.5745	0.5974	0.5101	0.5168	0.8741
Tab-Transformer	0.5821	0.4936	0.5003	0.5798	0.5195	0.4936	0.4370	0.4405	0.8091
CNN-1D	0.5187	0.5097	0.4797	0.5333	0.4852	0.5097	0.3855	0.3912	0.8078
FT-Transformer (NEW)	0.7810	0.6864	0.6796	0.7671	0.7544	0.6864	0.7081	0.7110	0.9188
NODE (NEW)	0.6455	0.5828	0.5725	0.6468	0.5701	0.5828	0.5349	0.5376	0.8716

**Table 6:** Per-class classification report for DNN (5-layer) (best DL model), 5-class task. Test set support: CN =62, SMC =84, EMCI =63, LMCI =122, AD =16. The AD class shows the most critical failure: recall of only 0.12.

Class	Precision	Recall	F1-Score	Support
CN	0.77	0.81	0.79	62
SMC	0.69	0.73	0.71	84
EMCI	0.64	0.78	0.71	63
LMCI	0.79	0.70	0.74	122
AD	0.22	0.12	0.16	16
Macro avg	0.62	0.63	0.62	347
Weighted avg	0.71	0.71	0.71	347

**Table 7:** Hybrid ensemble performance for all weight configurations. All evaluations on the official held-out test set (Test_data = 1). *w* denotes the LightGBM weight; 1−w is the DNN weight. Row ① is the ML-only baseline. Note: Row ⑤ (w=0.685) is a post hoc analytical upper bound derived by exhaustive sweep on the held-out test labels and does not constitute a valid generalisation result. All performance claims are based on Row ④ ($w^{★}=0.5024$, val-optimised).

Configuration	*w*	Accuracy	Bal. Acc.	Macro F1	Wt. F1	Cohen’s κ	MCC	ROC-AUC
① Pure LightGBM (ML baseline)	1.000	0.8156	0.7027	0.6906	0.8017	0.7537	0.7558	0.9310
② Pure DNN (5-layer) (DL baseline)	0.000	0.7118	0.6264	0.6194	0.7066	0.6178	0.6193	0.8940
③ Equal Weights	0.500	0.8069	0.6955	0.6832	0.7953	0.7428	0.7448	0.9236
④ GA Hybrid ($w^{★}=0.5024$, val-optimised)	0.502	0.8069	0.6955	0.6832	0.7953	0.7428	0.7448	0.9237
⑤ Best Hybrid (w=0.685, test-sweep)	0.685	0.8184	0.7067	0.6933	0.8056	0.7581	0.7606	0.9259

**Table 8:** Absolute metric improvement (Δ) of each hybrid configuration relative to the pure LightGBM baseline (row ①).

Configuration	Δ Acc.	Δ Bal. Acc.	Δ Macro F1	Δ Wt. F1	Δ Cohen’s κ	Δ MCC
① Pure LightGBM (baseline)	0.0000	0.0000	0.0000	0.0000	0.0000	0.0000
② Pure DNN (5-layer)	−0.1037	−0.0763	−0.0713	−0.0950	−0.1359	−0.1365
③ Equal Weights	−0.0087	−0.0072	−0.0075	−0.0064	−0.0109	−0.0110
④ GA Hybrid ($w^{★}=0.5024$)	−0.0086	−0.0072	−0.0075	−0.0064	−0.0109	−0.0110
⑤ Best Hybrid (w=0.685)	+0.0029	+0.0040	+0.0027	+0.0039	+0.0043	+0.0047

**Table 9:** Ablation study: GA-optimised hybrid performance for all four ML × DL combinations (n=347). Original configuration marked with ^†^.

ML	DL	$w^{★}$	Accuracy	Macro-F1	Cohen’s κ	MCC
LightGBM ^†^	DNN (5-layer) ^†^	0.550	0.7954	0.6744	0.7270	0.7288
LightGBM	FT-Transformer	0.385	0.8040	0.6817	0.7379	0.7394
XGBoost	DNN (5-layer)	0.562	0.7781	0.6750	0.7042	0.7056
XGBoost	FT-Transformer	0.482	0.8012	0.6798	0.7346	0.7365

**Table 10:** Federated Hybrid Ensemble: test-set performance of FL-DNN + centralised LightGBM ($w^{★}=0.5024$) for all four FL algorithms. Sorted by accuracy.

FL Algorithm	Accuracy	Bal. Acc.	Macro F1	Wt. F1	Prec.	Recall	Cohen’s κ	MCC	ROC-AUC
FedNova	0.8213	0.7076	0.6953	0.8073	0.7241	0.7076	0.7614	0.7634	0.9241
FedAvg	0.8069	0.6901	0.6829	0.7934	0.7144	0.6901	0.7410	0.7422	0.9072
FedProx	0.8040	0.6869	0.6804	0.7906	0.7126	0.6869	0.7369	0.7381	0.9092
SCAFFOLD	0.7579	0.6164	0.6185	0.7407	0.6317	0.6164	0.6673	0.6742	0.8747

**Table 11:** Absolute gain (Δ) of the federated hybrid ensemble over the FL-DNN alone for each algorithm. All Δ values are positive, confirming that the centralised LightGBM component consistently improves the federated DNN.

Algorithm	Accuracy		Δ Acc.	Macro F1		Δ F1	Cohen’s κ	
	DNN	Hybrid		DNN	Hybrid		DNN	Hybrid
FedNova	0.4063	0.8213	+0.415	0.3198	0.6953	+0.376	0.2491	0.7614
FedAvg	0.4352	0.8069	+0.372	0.2493	0.6829	+0.434	0.1628	0.7410
FedProx	0.4380	0.8040	+0.366	0.2548	0.6804	+0.426	0.1682	0.7369
SCAFFOLD	0.3516	0.7579	+0.406	0.1041	0.6185	+0.514	0.0000	0.6673

**Table 12:** Grand summary: Best representative result from each method category, ranked by accuracy.

Model	Category	Accuracy	Bal. Acc.	Macro F1	Cohen’s κ	MCC	ROC-AUC
FedNova Hybrid	FL + Hybrid	0.8213	0.7076	0.6953	0.7614	0.7634	0.9241
Best Hybrid (w=0.685)	Hybrid	0.8184	0.7067	0.6933	0.7581	0.7606	0.9259
LightGBM	ML	0.8156	0.7027	0.6906	0.7537	0.7558	0.9310
XGBoost	ML	0.8040	0.7040	0.6977	0.7391	0.7409	0.9400
GA Hybrid ($w^{★}=0.502$)	Hybrid	0.8069	0.6955	0.6832	0.7428	0.7448	0.9237
FedAvg Hybrid	FL + Hybrid	0.8069	0.6901	0.6829	0.7410	0.7422	0.9072
FedProx Hybrid	FL + Hybrid	0.8040	0.6869	0.6804	0.7369	0.7381	0.9092
DNN (5-layer)	DL	0.7118	0.6264	0.6194	0.6178	0.6193	0.8940
SCAFFOLD Hybrid	FL + Hybrid	0.7579	0.6164	0.6185	0.6673	0.6742	0.8747

## Data Availability

The dataset used in this study is publicly available on the Kaggle platform at: https://www.kaggle.com/datasets/sarthakkanjariya/alzheimer-dataset?resource=download (accessed on 12 January 2026). This is a preprocessed tabular CSV file accessible without registration or data use agreement. No direct access to the ADNI repository was used in this study.
